# MicroRNA-155 Inhibition Activates Wnt/β-Catenin Signaling to Restore Th17/Treg Cell Balance and Protect against Acute Ischemic Stroke

**DOI:** 10.1523/ENEURO.0347-24.2024

**Published:** 2025-02-18

**Authors:** Wenli Huang, Quanlong Hong, Huimin Wang, Zhihua Zhu, Shujie Gong

**Affiliations:** Department of Neurology, Quanzhou First Hospital Affiliated to Fujian Medical University, Quanzhou 362002, China

**Keywords:** bioinformatics, ischemic stroke, miR-155, Th17/Treg, Wnt/β-catenin

## Abstract

Acute ischemic stroke (AIS) is a severe neurological disease associated with Th17/Treg cell imbalance and dysregulation of the Wnt/β-catenin signaling pathway. This study investigates whether miR-155 inhibition can activate Wnt/β-catenin signaling, improve Th17/Treg balance, and provide neuroprotection against stroke. We conducted a multilevel experimental design, including high-throughput sequencing, bioinformatics analysis, in vivo mouse models, and in vitro cell experiments. High-throughput sequencing revealed significant differential gene expression between the miR-155 antagomir–treated and control groups (BioProject: PRJNA1152758). Bioinformatics analysis identified key genes linked to Wnt/β-catenin signaling and Th17/Treg imbalance. In vitro experiments confirmed that miR-155 inhibition activated Wnt/β-catenin signaling and improved Th17/Treg ratios. In vivo studies demonstrated that miR-155 antagomir treatment provided significant neuroprotection against AIS. These findings suggest that targeting miR-155 could be a promising therapeutic strategy for stroke by modulating immune balance and key signaling pathways.

## Significance Statement

This study identifies miR-155 as a pivotal regulator of the T helper 17/regulatory T-cell balance and Wnt/β-catenin signaling pathway in acute ischemic stroke. By inhibiting miR-155, we demonstrate the potential to enhance neuroprotection and modulate immune responses, offering a promising therapeutic avenue for stroke management. These findings contribute to the growing understanding of molecular mechanisms in stroke and provide a foundation for developing miR-155–targeted therapies.

## Introduction

Acute ischemic stroke (AIS) is a common but serious neurological disorder with high incidence and mortality rates worldwide (Ma et al., 2022; H. Lu et al., 2023; Z. Lu et al., 2023; Zeng et al., 2023). Studies have shown a close correlation between AIS and abnormal immune reactions and disrupted signaling pathways of the immune system (Gong et al., 2021; Qiu et al., 2021; Cai et al., 2023). Specifically, an imbalance in T helper 17 (Th17)/regulatory T- (Treg) cells and abnormal activation of the Wnt/β-catenin signaling pathway are considered critical mechanisms in AIS (Zhang et al., 2021a,b; Shan et al., 2023). However, the understanding of these two mechanisms and their interactions is still incomplete (Wu et al., 2021; Wang et al., 2022). Therefore, a deep investigation into the relationship between these two mechanisms is of great significance for the development of more effective stroke treatment strategies.

Th17 cells are a subset of T-cells primarily secreting proinflammatory factors like interleukin (IL)-17 and IL-22, differentiated from naive T-cells under the stimulation of IL-6 and transforming growth factor (TGF)-β. The transcription factor retinoic acid-related orphan receptor γ (RORγ) is preferentially expressed in Th17 cells and is essential for their differentiation and development (Cyr et al., 2016; Basu et al., 2021). Conversely, Treg cells serve as anti-inflammatory regulators, producing IL-10, TGF-β, and other cytokines upon TGF-β stimulation. Studies have shown that proinflammatory cytokines are mainly produced by Th cells, especially Th17 cells, while anti-inflammatory cytokines are primarily produced by Treg cells (Strober et al., 2010; Qiu et al., 2013).

The Wnt/β-catenin signaling pathway, also known as the canonical Wnt pathway, is a conserved axis involved in various physiological processes, including proliferation, differentiation, apoptosis, migration, invasion, and tissue homeostasis (Choi et al., 2020; Salik et al., 2020). Prior studies have linked canonical Wnt/β-catenin signaling to cerebrovascular development and microvascular barrier properties (Stenman et al., 2008; Daneman et al., 2009). However, the endoluminal signal of β-catenin must be tightly regulated, as prolonged high levels of β-catenin in blood vessels can significantly impact vascular stability and lumen morphology (Corada et al., 2010).

miRNAs, as a class of noncoding RNA molecules with important regulatory functions, have been proven to play a crucial role in various diseases (Li et al., 2021; Hashemi et al., 2022; Sinha et al., 2022). miR-155, a widely studied miRNA, plays an important role in immune response and inflammatory processes (Greco et al., 2022; Moutabian et al., 2023; Xue et al., 2023). Previous studies have indicated that miR-155 regulates the Wnt/β-catenin signaling pathway in diseases such as osteosarcoma, familial adenomatous polyposis, and periodontitis (Sun et al., 2015; Wang et al., 2017; Prossomariti et al., 2018). Furthermore, one study reported that miR-155 antagomir could activate Wnt/β-catenin and modulate the Th17/Treg cell balance (Zhu et al., 2020). Activation of the Wnt/β-catenin signaling pathway helps maintain blood–brain barrier (BBB) integrity, reduce the risk of parenchymal hemorrhage, and improve cerebral microvascularization, thereby providing neuroprotection in AIS (Ta et al., 2021; Liu et al., 2022; Tian et al., 2022). These findings highlight miR-155’s importance in AIS pathology, though its precise mechanism remains unclear. This study aims to explore the effects of miR-155 inhibition on the Wnt/β-catenin pathway and the Th17/Treg cell balance, assessing its potential neuroprotective impact against AIS.

## Materials and Methods

### Ethical statement

All animal experiments were approved by the Animal Ethics Committee of Quanzhou First Hospital Affiliated to Fujian Medical University. The study was carried out in strict accordance with the approved guidelines and regulations, ensuring the ethical treatment of all animals used in the research.

### Mouse model of middle cerebral artery occlusion (MCAO)

Male C57BL/6 mice aged 8–10 weeks were obtained from Vital River Laboratories (101). To begin, we anesthetized the mice with 3% isoflurane (Sigma-Aldrich, catalog #792632). The mice were then positioned in a supine position with their limbs and head secured. The right common carotid artery (CCA), external carotid artery (ECA), and internal carotid artery were exposed to facilitate the occlusion of the middle cerebral artery (MCA). The proximal end of the CCA was ligated with silk suture, and a small incision was made at the distal end of the ECA. A nylon suture (18 mm long, Xinhong Technology, catalog #2634A4) was inserted into the ECA and advanced through to reach the MCA. The nylon suture was pushed until encountering mild resistance, at which point it was halted to mimic the occlusion. After a 2 h period of occlusion, the nylon suture was subsequently withdrawn.

The same surgical procedure for vascular dissection was performed on the mice in the sham-operated group, except for the occlusion step. Throughout the entire experiment, the mice's body temperature was maintained at 37°C (Yang et al., 2011; Xu et al., 2023).

### Intracerebral injection pretreatment

Two hours prior to performing MCAO surgery on mice, intracerebral injections were administered. The coordinates for injection were determined using a stereotaxic instrument (Stoelting) at the ipsilateral hemisphere region (0.2 mm anterior to the bregma, 1.0 mm lateral to the midline, and 1.5 mm below the brain surface). Antagomir (3 pmol/g, final volume 2 μl per mouse) was mixed with IWP-2-liposomes at 4 μl per mouse (MCE, HY-13912) and injected 2 h later (Xu et al., 2015). The mice were divided into the following groups (*n* = 10 per group): (1) sham group (sham surgery); MCAO group; MCAO + miR-155 antagomir (miR-155 antagomir pretreatment); MCAO + antagomir NC (antagomir NC pretreatment); MCAO + miR-155 antagomir + DMSO (miR-155 antagomir pretreatment with intraperitoneal DMSO injection); MCAO + antagomir NC + DMSO (antagomir NC pretreatment with intraperitoneal DMSO injection); and MCAO + miR-155 antagomir + IWP-2 (miR-155 antagomir combined with IWP-2 pretreatment). The sequence for miR-155-3p is 5′-CTCCUACCUGUGUAUAUAC-3′, and the sequence for the NC is 5′-CAGUACUUUUGUGUAGUACAA-3′.

### Preparation of blood and serum

Blood samples were collected via cardiac puncture using a syringe without the need for open-chest surgery. For serum samples, the blood samples were centrifuged at 4,000 rpm for 4 min at 4°C, and the supernatant was immediately transferred and stored at −80°C for later use (Jin et al., 2017).

### Neurological function assessment

Neurological function assessment was conducted using the Zea–Longa five-point scale: 4, inability to walk spontaneously and loss of consciousness; 3, leaning to the opposite side while walking; 2, rotating to the opposite side while walking; 1, incomplete extension of the contralateral forepaw; 0, no symptoms of injury, with higher scores indicating more severe damage (PMID, 26522298; PMCID, PMC6493568).

### 2,3,5-Triphenyltetrazolium Chloride (TTC) staining

After 24 h of MCAO surgery, mice were killed, and the brain tissue was harvested to make 2 mm brain tissue sections. The sections were immediately placed in a 2% TTC solution (G3005, Solarbio), incubated at 37°C in the dark for 30 min, and then fixed with 4% paraformaldehyde for 2 h. The brain tissue sections were scanned to obtain images, and the ImageJ software was used to calculate the area of cerebral infarction. The volume of brain infarction (%) was calculated using the following formula: (volume of the contralateral hemisphere − volume of the unaffected hemisphere) / (volume of the contralateral hemisphere × 2) (Clarkson et al., 2014; Wu et al., 2018; Xie et al., 2019).

### Measurement of brain water content

Twenty-four hours after MCAO surgery, mice were killed, and the brain tissue was immediately extracted within 1 min. The wet weight of the brain tissue was then measured and placed at 100°C for 24 h to obtain the dry weight. Brain edema was determined using the following formula: the percentage of brain water content = (wet weight − dry weight) / wet weight × 100% (Yang et al., 2011; Xu et al., 2023).

### H&E staining

The brain tissue was fixed in 4% paraformaldehyde and then subjected to gradient ethanol dehydration, transparency, and routine paraffin embedding. Serial sections with a thickness of 4 µm were cut using a paraffin microtome, followed by baking at 60°C for 1 h and deparaffinization with xylene. Routine H&E staining was performed by staining with hematoxylin for 10 min and eosin for 2 min. The tissue morphology was observed under an optical microscope (XP-330, Shanghai Bingyu Optical Instrument; Wu et al., 2018; Xu et al., 2023).

### Nissl staining

Using the same paraffin sections mentioned above, deparaffinization was done in xylene, followed by ethanol dehydration. Following the manufacturer's instructions, Nissl staining reagent (C0117, Beyotime Biotech) was used. In brief, the sections were incubated in Nissl staining solution at 50°C for 5 min, washed twice with distilled water, and subjected to gradient ethanol washes. The observation was conducted under a fluorescence microscope (Olympus IX73; Jiang et al., 2021).

### TUNEL staining

To perform TUNEL staining, the paraffin-embedded sections were deparaffinized in xylene and dehydrated using a graded ethanol series. Cell apoptosis was detected using the TUNEL staining kit (C1086, Beyotime Biotech). In brief, the sections were incubated with a mixture containing 50 μl of TUNEL reaction mixture, which consisted of 45 μl of labeling buffer and 5 μl of TdT enzyme solution. The reaction mixture was then incubated for 1 h and visualized under a fluorescence microscope (Huang and Wang, 2023).

### Measurement of BBB permeability

The integrity and permeability of the BBB were quantitatively assessed by measuring the extravasation of the Evans blue dye, a marker of albumin leakage. Two hours after MCAO surgery, mice were intravenously injected with a 2% solution of Evans blue dye (24 ml/kg, Solarbio) via the tail vein. After 2 h, the remaining dye in the vasculature was cleared by perfusing the heart with saline. The brain hemispheres were weighed and then placed in dimethylformamide (Sigma-Aldrich) overnight at 60°C. The absorbance was measured at 632 nm, and a standard curve was established using a gradient concentration (Wang et al., 2016; H. Lu et al., 2023; Z. Lu et al., 2023).

### Immunofluorescence staining

Cells were fixed with 4% paraformaldehyde, permeabilized with 0.1% Triton X-100, and incubated overnight with NeuN antibody (ab104224, 1:1,000, Abcam) and Foxp3 antibody (PA1-46126, 1:500, Thermo Fisher Scientific) at 4°C. After washing with PBS three times, the sections were incubated for 1 h at 37°C with Alexa Fluor 488-conjugated goat anti-mouse IgG (ab150117, 1:1,000, Abcam) and Alexa Fluor 594-conjugated goat anti-rabbit IgG (ab150080, 1:1,000, Abcam). The sections were observed under a fluorescence microscope (Liang et al., 2019).

### Primary cultures of mouse cortical neurons

For the generation of primary cultures of mouse cortical neurons, we obtained Embryonic Day 18 (E18) C57BL/6 mouse brains. The cortex tissue was dissected into small pieces using a sterile surgical blade. The tissue was then digested at 37°C for 20 min using 0.25% trypsin (25200-072, Thermo Fisher Scientific). Enzyme activity was stopped by adding Dulbecco's Modified Eagle Medium (DMEM; 11885092, Thermo Fisher Scientific) supplemented with 10% fetal bovine serum (FBS; 12484028, Thermo Fisher Scientific). The cells were then seeded onto six-well culture plates with poly-L-lysine (100 μg/ml) coating. After 4 h of incubation, the culture medium was replaced with Neurobasal medium supplemented with 2% B-27 and 1% L-glutamine (Neurobasal medium, 21103049; Thermo Fisher Scientific; B-27 supplement, 17504-044, Thermo Fisher Scientific; L-glutamine, 35050-061, Thermo Fisher Scientific). The medium was half-replaced every day (Yao et al., 2019). Neurons were identified using immunostaining with class III β-tubulin (1:100, ab18207, Abcam) and Hoechst 33342 (14533, Sigma-Aldrich; Lai et al., 2020).

### Oxygen–glucose deprivation/reoxygenation (OGD/R) model

After 7 d of neuronal culture, we established the OGD/R model. Initially, neurons were placed in an anaerobic chamber containing 5% CO_2_ and 95% N_2_ at 37°C. They were cultured for 4 h in glucose-free DMEM (11966-025, Thermo Fisher Scientific) supplemented with 10% FBS. Subsequently, the medium was switched back to the normal culture medium, and the neurons were maintained at 37°C in a 5% CO_2_ incubator for 12 h for reoxygenation. The control group cells were cultured under normal conditions with a complete medium (Scorziello et al., 2007; Chen et al., 2016). To transfect the cells, we employed Lipofectamine 3000 transfection reagent (L3000001, Thermo Fisher Scientific) to mediate the transfection of miR-155 inhibitor (MCE). The transfected cells were then cultured for 48 h. For further analysis, some groups were supplemented with 20 μM IWP-2 (dissolved in PBS, MCE, HY-13912) after 48 h of cell culture. The cell groups were as follows: control group, OGD/R group, OGD/R + miR-155 inhibitor group (OGD/R-induced miR-155 knockdown), and OGD/R + miR-155 inhibitor + IWP-2 group (OGD/R-induced miR-155 knockdown with IWP-2 treatment).

### Sequencing and initial data processing

Neuronal cells were extracted from the control group and the OGD/R group using TRIzol reagent, following the manufacturer's instructions. The precipitated RNA was then dissolved in DEPC-treated water. The concentration and purity of the RNA were measured using a NanoDrop, ensuring a 260/280 ratio between 1.8 and 2.1. To assess the integrity of the RNA, we used Agilent Bioanalyzer, with a requirement for an RNA integrity number >7. An rRNA removal kit was employed to eliminate rRNA from the samples. Library preparation was accomplished using NEB or Illumina kits through random RNA fragmentation followed by the addition of sequencing adaptors. The quality and size of the library products were verified using Qubit and Agilent Bioanalyzer.

High-throughput sequencing was conducted on an appropriate platform such as Illumina HiSeq or NovaSeq, and the raw data have been deposited in the SRA under BioProject PRJNA1152758 with the following SRA IDs: SRR30410532, SRR30410531, and SRR30410530 (disease group) and SRR30410529, SRR30410528, and SRR30410527 (control group). After sequencing, the quality of the raw data was examined using FastQC, ensuring that the Q30 value exceeded 90%. The Trim Galore or Trimmomatic software was used for read trimming, eliminating low-quality reads and Illumina adaptors. Processed reads were aligned to a reference genome using the HISAT2 or STAR software, with a requirement for an alignment efficiency above 90%.

Differential gene expression analysis was performed on the dataset using the “limma” package in R. The filtering criteria included |logFC| > 1 and *p* < 0.05. A heat map depicting differential gene expression was generated using the “heatmap” package, and a volcano plot illustrating gene expression differences was created using the “ggplot2” software package. All analyses were conducted in R version 4.2.1 (R Foundation for Statistical Computing Liu et al., 2018).

### GO and KEGG functional enrichment analysis

The differentially expressed genes were subjected to Gene Ontology (GO) and Kyoto Encyclopedia of Genes and Genomes (KEGG) functional enrichment analysis using R packages such as “clusterProfiler,” “org.Hs.eg.db,” “enrichplot,” and “GOplot.” Additionally, bubble plots were generated to visualize the results of the GO and KEGG functional enrichment analysis (Zhang et al., 2018).

### Dual-luciferase reporter gene assay

The TargetScan website predicted the binding site between miR-155-3p and Wnt2b. To investigate this interaction, the pMIR vector (AM5795, Thermo Fisher Scientific) was constructed to generate wild-type (WT) and mutant (MUT) Wnt constructs. The luciferase reporter gene vectors, along with mmu-miR-155 antagomir, were cotransfected into neuronal cells using Lipofectamine 3000. After 24 h, quantitative fluorescence values were measured using the Dual-Luciferase Reporter Assay Kit (HY-K1013, MCE), and data analysis was performed using the SpectraMax M5 software (Molecular Devices; Zhao et al., 2020).

### Treg cell isolation

Single-cell suspensions from fresh spleens of C57BL mice were prepared, and Treg cells were enriched using a negative selection and Treg cell isolation kit (130-092-984, Miltenyi Biotec) following the manufacturer's instructions. Treg cells were labeled with anti-CD4 and CD25 antibodies and then coupled with magnetic beads. Magnetic cell separation was performed by passing the cell suspension through a column containing a magnetic metal matrix to separate the labeled cells. Non-CD4 cells were first removed, followed by the isolation of CD25-positive cells from the CD4-enriched solution, resulting in the Treg cell population of CD4/CD25 cells (Borlongan et al., 2021).

### Coculture of Treg cells and neurons

As previously mentioned, Treg cells were isolated from mouse splenic tissue and cocultured with neurons using the following procedure. First, the culture medium was removed from the semifused neuron cell culture, and Treg cells were added (1.0 × 10^6^/well). Preactivated Treg cells were obtained by stimulating them with CD3/CD28 antibodies (11452D, Thermo Fisher Scientific) and cocultured for 3 d. Subsequently, the Treg cells were seeded into the wells of a 96-well Transwell coculture system (SPL Life Sciences) without antibodies. Simultaneously, primary cultured neurons (2 × 10^5^/well) were subjected to a 4 h OGD induction treatment (the control group was not subjected to OGD induction) and then added to the 96-well Transwell coculture system. The cells were cultured at 37°C for 24 h in RPMI 1640 medium supplemented with 10% FBS, 100 U/ml penicillin, and 100 μg/ml streptomycin (C. Wang et al., 2023; S. Wang et al., 2023).

### Flow cytometry

For in vivo experiments, peripheral blood mononuclear cells were isolated and prepared as described earlier. Flow cytometry was performed to determine the percentages of Th17 and Treg cells, as well as the Th17/Treg cell ratio. The antibodies used in the experiment were FITC-conjugated CD4 (11-0041-82, Thermo Fisher Scientific), APC-conjugated CD25 (12-0251-82, Thermo Fisher Scientific), PE-conjugated Foxp3 (12-5773-82, Thermo Fisher Scientific), and PE-conjugated IL-17 (12-7182-82, Thermo Fisher Scientific). Data acquisition was performed using a flow cytometer (BD Biosciences; Zhang et al., 2021a,b).

### Apoptosis detection

Cell apoptosis was detected using the Annexin V-FITC/PI assay kit (C1062L, Beyotime Biotech). Initially, cells were collected and washed with PBS, followed by resuspension in the Annexin V-FITC buffer. Then, 5 µl of Annexin V/FITC and 10 µl of PI were added and incubated in a light-protected environment at room temperature for 15 min. Finally, analysis was performed using the FACScan flow cytometer (BD Biosciences; Fan and Zhou, 2021). The apoptotic cell percentage was calculated as the sum of cells in the Q1-UR (upper right quadrant) and Q1-LR (lower right quadrant; Chen et al., 2021).

### Enzyme-linked immunosorbent assay (ELISA)

Mouse serum was used to evaluate circulating proinflammatory cytokines, namely, IL-6 (PI326, Beyotime Biotech), IL-17 (PI545, Beyotime Biotech), TNF-α (PT512, Beyotime Biotech), IL-10 (PI522, Beyotime Biotech), and TGF-β (PT878, Beyotime Biotech), using the ELISA technique. As per the instructions provided with the assay kits, absorbance (OD) at 450 nm was recorded using a microplate reader (Bio-Rad Laboratories; Zhang et al., 2021a,b).

### Real-time quantitative polymerase chain reaction (RT-qPCR)

The tissue (ipsilateral ischemic cerebral cortex) and total RNA from cells were extracted using the Trizol reagent kit (10296010, Thermo Fisher Scientific). Reverse transcription was then performed using the PrimeScript RT-qPCR kit (RR086A, Takara Bio). The SYBR Premix Ex TaqTM II kit (DRR081, Takara Bio) was used to prepare the reaction system, which was subsequently subjected to real-time quantitative reverse transcription polymerase chain reaction (qRT-PCR) using the ABI 7500 real-time fluorescence quantitative PCR instrument (Thermo Fisher Scientific). GAPDH was used as an internal control, and three replicates were set for each RT-qPCR experiment. The primers for amplification were provided by Shanghai Biotech, and the primer sequences can be found in Table 1. The fold change in gene expression between the experimental and control groups was determined using the 2^−ΔΔCt^ method, where ΔΔCT = ΔCt_experimental group_ − ΔCt_control group_ and ΔCt = Ct_target gene_ − Ct_internal reference gene_. Ct represents the number of amplification cycles required for the real-time fluorescent intensity to reach the preset threshold (Ayuk et al., 2016). Each experiment was repeated three times.

**Table 1.** RT-qPCR primer sequence (mouse)

Gene name	Primer sequence
IL-10	F: 5′-AGGCGCTGTCATCGATTTCT-3′
	R: 5′-ATGGCCTTGTAGACACCTTGG-3′
IL-17	F: 5′-AGAAGTTCCCAAATGGCCTC-3′
	R: 5′-CCACTTGGTGGTTTGCTACG-3′
IL-6	F: 5′-AGTTGCCTTCTTGGGACTGA-3′
	R: 5′-TCCACGATTTCCCAGAGAAC-3′
TGF-β	F: 5′-CGTGGAAATCAACGCTCCAC-3′
	R: 5′-CCACGTAGTAGACGATGGGC-3′
Mmu-miR-155-3p	F: 5′-CTCCTACC TGTTAGCATTAAC-3′
	Universal primers
GAPDH	F: 5′-AAGATGGTGAAGGTCGGTG-3′
	R: 5′-GTTGATGGCAACAATGTCCAC-3′

F, forward; R, reverse.

### Western blot

Protein extraction was performed using the Bestbio Protein Extraction Kit (BB3101) to extract total proteins from cells or tissues. Cell lysis was carried out using an enhanced RIPA lysis buffer (AR0108, Wuhan Bodun Technology) containing proteinase inhibitors. Protein concentrations were determined using the BCA Protein Quantification Kit (AR1189, Wuhan Bodun Technology). A 10% SDS–PAGE gel (Beyotime Biotech, P0012A) was prepared, and each well was loaded with 50 μg of protein samples. The gel was subjected to constant voltage electrophoresis at 80–120 V for 2 h. The proteins were then transferred to a PVDF membrane (Merck Millipore, IPVH00010) at a constant current of 250 mA for 90 min. The PVDF membrane was blocked with a 5% nonfat milk TBST solution at room temperature for 4 h, followed by three washes with TBST. The primary antibody (shown in Table 2) was diluted in 5% BSA (9048-46-8, Solarbio) and incubated overnight at 4°C, followed by three washes with TBST for 10 min each. The membrane was then incubated with either the anti-mouse-HRP secondary antibody [catalog #7076, 1/5,000, Cell Signaling Technology (CST)] or the anti-rabbit-HRP secondary antibody (catalog #7074, 1/5000, CST) at room temperature for 1 h, followed by three washes with TBST for 10 min each. Protein bands were visualized using the ECL detection kit (Beyotime Biotech, P0018FS), and the membrane was exposed and developed in a dark box. Each sample was replicated in three independent experiments. The ImageJ analysis software was used for quantitative analysis of the Western blot bands, with GAPDH serving as the internal control (J. Lu et al., 2018; Y. Lu et al., 2018; Luo et al., 2019).

**Table 2.** Details of primary antibody

Name	Catalog	Species	Dilution ratio	Manufacturer	Country
ROR-γt	14-6988-82	Human and mouse	1: 100	Thermo Fisher Scientific	USA
β-Catenin	MA5-34961	Human and mouse	1: 500	Thermo Fisher Scientific	USA
Wnt2b	710888	Human and mouse	1: 500	Thermo Fisher Scientific	USA
Foxp3	PA1-46126	Human and mouse	1: 2,000	Thermo Fisher Scientific	USA
GADPH	SAB4300645	Human and mouse	1:1,000	Sigma-Aldrich	USA

### CCK-8 assay

Cell viability was measured using the CCK-8 assay kit (ab228554, Abcam) as per the manufacturer's instructions. Cells from each group were seeded in 96-well plates at a density of 1 × 10^4^ cells per well (200 μl/well). After 48 h of incubation, 10 μl of the CCK-8 reagent was added to each well. Following a 2 h incubation period, absorbance was measured at 450 nm using a microplate reader (M1000 PRO, Tecan; Zhou et al., 2021).

### Statistical analysis

The sample size was determined using the G*Power version 3.1 software. An a priori power analysis was conducted for an ANCOVA with fixed effects, main effects, and interactions, with the following parameters: effect size *f* = 0.4; α error probability, 0.05; power (1 − β) = 0.9; number of groups = 7. The analysis indicated a total sample size requirement of 138 mice, resulting in ∼20 mice per group to achieve a power of 0.901 with the given parameters.

The statistical analysis software used in this study included GraphPad Prism 8 (version 8.0.2.263, GraphPad Software) and SPSS 21.0 software (SPSS). For animal experiments, each group consisted of 20 mice (5 for brain water content measurement, 5 for BBB permeability experiments, and 10 for other experiments). Cell experiments were repeated three times. The Shapiro–Wilk test was used to assess the normality of data distribution. Measurement data were expressed as mean ± standard deviation, and comparisons between two groups were performed using unpaired *t* tests. Comparisons among multiple groups were conducted using one-way analysis of variance. Nonparametric tests were used for data with non-normal distribution. Categorical data were expressed as rates or percentages and were analyzed using the *χ*^2^ test. A *p* value of <0.05 was considered statistically significant.

## Results

### Differential gene expression and functional enrichment analysis in ischemic brain injury

**Figure 1.**
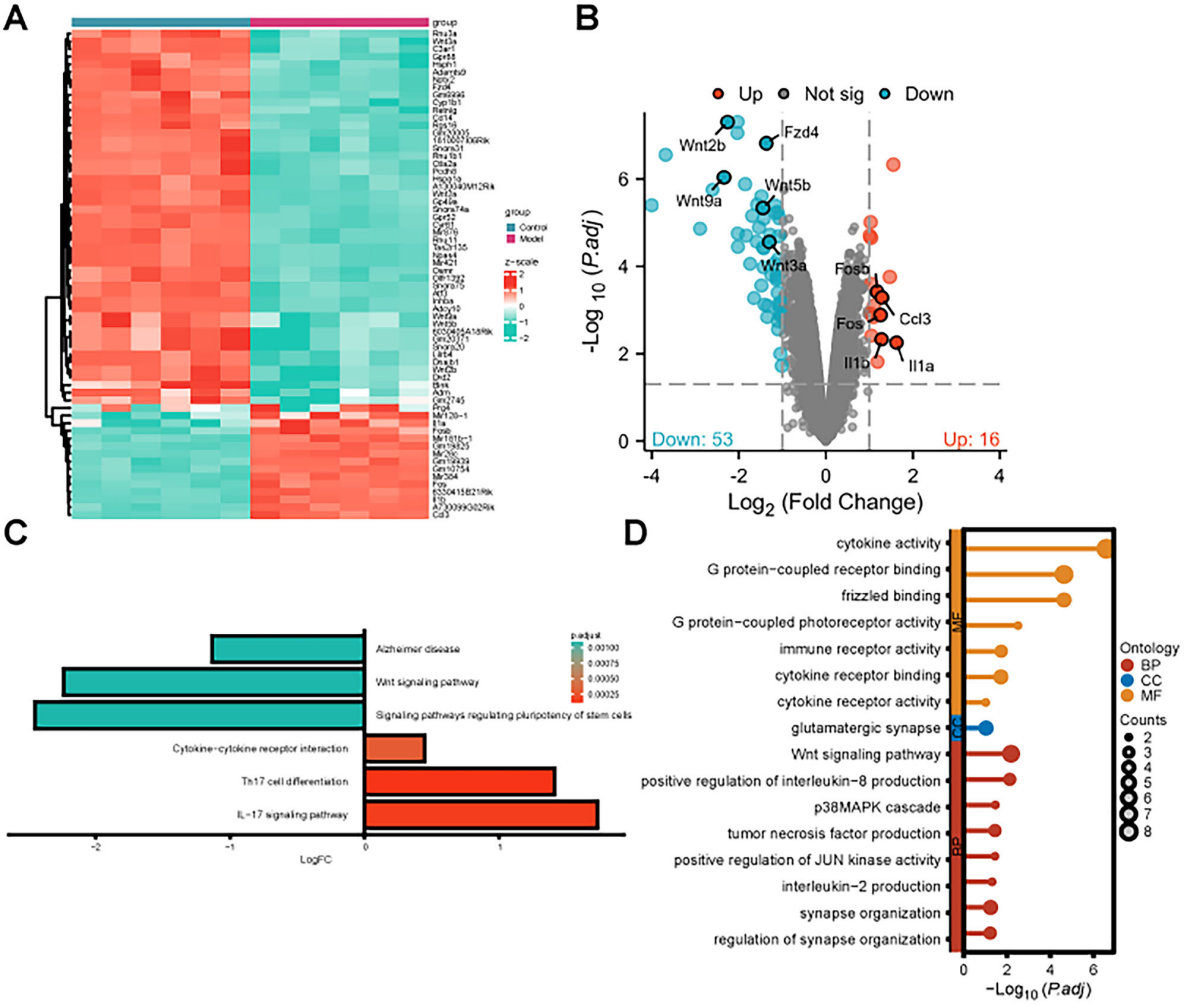
Differential gene expression analysis of neurons and OGD/R neurons. ***A***, A volcano plot of differentially expressed genes in the dataset: control, *n* = 3; model, *n* = 3; ***B***, Heatmap of differentially expressed genes in the dataset; ***C***, KEGG pathway enrichment analysis of intersecting genes; ***D***, GO functional enrichment analysis of intersecting genes in BP, CC, and MF.

To investigate the crucial factors influencing ischemic brain injury, we performed RNA-seq on neurons subjected to OGD/R induction. We obtained a dataset and conducted differential gene expression analysis. In total, we identified 69 differentially expressed genes, represented in a heatmap (Fig. 1*A*), with 16 upregulated and 53 downregulated genes (Fig. 1*B*). Furthermore, we performed GO and KEGG functional enrichment analyses on these differentially expressed genes. The KEGG pathway enrichment analysis revealed an enrichment of differentially expressed genes in inflammation-related pathways such as the Wnt and IL-17 signaling pathways (Fig. 1*C*). The GO functional analysis highlighted a higher enrichment in MF, including functions involving cytokine activity and G-protein–coupled receptor binding (Fig. 1*D*), indicating involvement in the activity of inflammatory cytokines.

### miR-155-3p affects ischemic brain injury by regulating the Wnt/β-catenin signaling pathway

**Figure 2.**
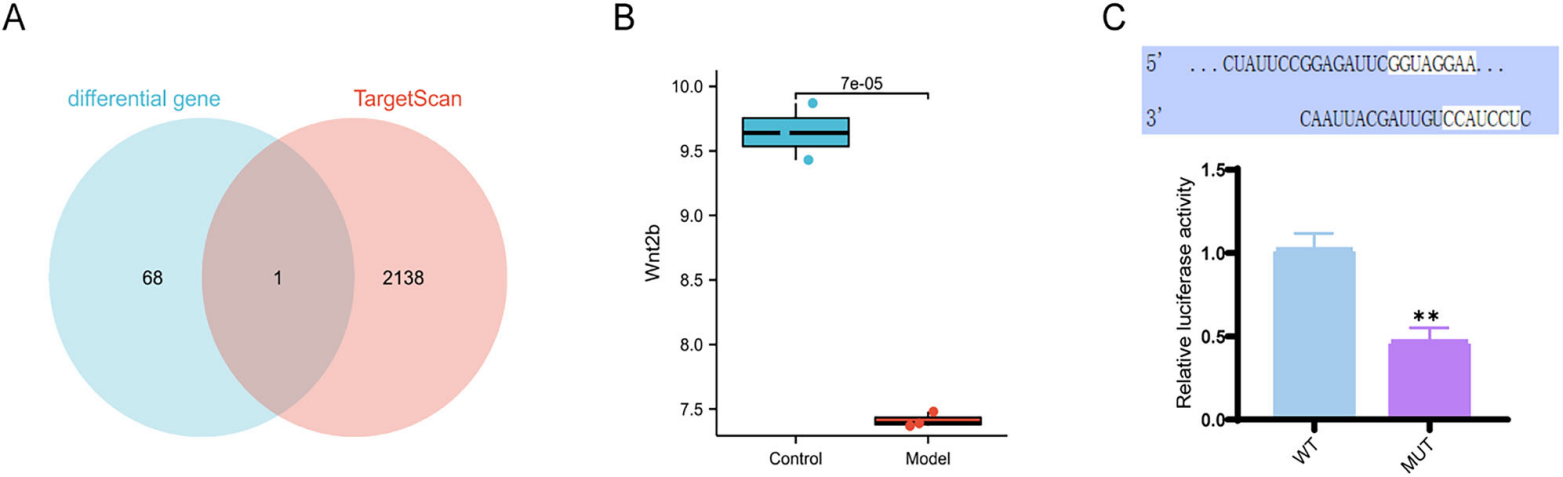
Targeting of Wnt2b gene by miR-155-3p. ***A***, A Venn diagram showing the intersection of differentially expressed genes and miR-155-3p interacting genes; ***B***, A box plot of differential expression of Wnt2b; ***C***, Dual-luciferase reporter gene assay confirmed the targeting relationship between mmu-miR-155-3p and Wnt2b, with * indicating a *p* value of <0.05 compared with the WT group; control group, *n* = 3; model group, *n* = 3.

In recent years, microRNAs have emerged as targets for the prevention and treatment of various human diseases. Studies have shown significant changes in the expression of multiple microRNAs in the tissue and organs affected by brain ischemia–reperfusion injury, indicating that microRNAs can directly or indirectly influence the damage caused by ischemia–reperfusion injury in the brain (Xiang et al., 2019). Recent studies have indicated that knocking out miR-155 can alleviate ischemia–reperfusion-induced brain injury and hemorrhagic transformation (Suofu et al., 2020). To investigate whether and how miR-155 affects ischemic brain injury, we predicted and downloaded potential target mRNAs of mmu-miR-155-3p through the TargetScan website, resulting in a total of 2,138 potential targets. Subsequently, we performed an intersection analysis between the differentially expressed genes and the potential target genes of miR-155-3p, yielding only one gene (Fig. 2*A*). Differential expression analysis revealed that Wnt2b showed a significant decrease in expression levels in the model group, as indicated by the boxplot with a *p* value of 7 × 10^−5^, which is <0.05 (Fig. 2*B*). To validate the interaction between mmu-miR-155-3p and Wnt2b, we constructed Wnt2b-pMIR plasmids with WT and MUT Wnt2b, and luciferase assay revealed significantly higher luciferase activity in the group cotransfected with mmu-miR-155-3p antisense RNA and WT-Wnt2b, indicating that mmu-miR-155-3p can directly interact with Wnt2b and inhibit its expression (Fig. 2*C*).

Therefore, these findings suggest that miR-155-3p may influence ischemic brain injury by acting on the Wnt/β-catenin signaling pathway.

### miR-155 antagomir alleviates ischemic brain injury by regulating the Wnt/β-catenin signaling pathway

Bioinformatics analysis suggests that miR-155 may influence ischemic brain injury through the Wnt/β-catenin signaling pathway. To validate this analysis, we first established a transient MCAO mouse model. Two hours before the surgery, we stereotactically injected miR-155 antagomir or control antagomir NC (Extended Data Fig. 3-1*A*). Subsequently, the expression levels of miR-155 in each group were assessed using qRT-PCR (Extended Data Fig. 3-1*B*). The results showed that, compared with the Sham group, miR-155 expression was significantly elevated in mice after MCAO surgery (*p* < 0.0001; 95% CI, −1.306 to −0.8156), indicating a potential role of miR-155 in ischemic brain injury. Furthermore, compared with the MCAO + antagomir NC group, miR-155 expression was significantly reduced in MCAO mice pretreated with miR-155 antagomir (*p* < 0.0001; 95% CI, 0.5056 to 0.9964), confirming the successful reduction of miR-155 by miR-155 antagomir pretreatment.

**Figure 3.**
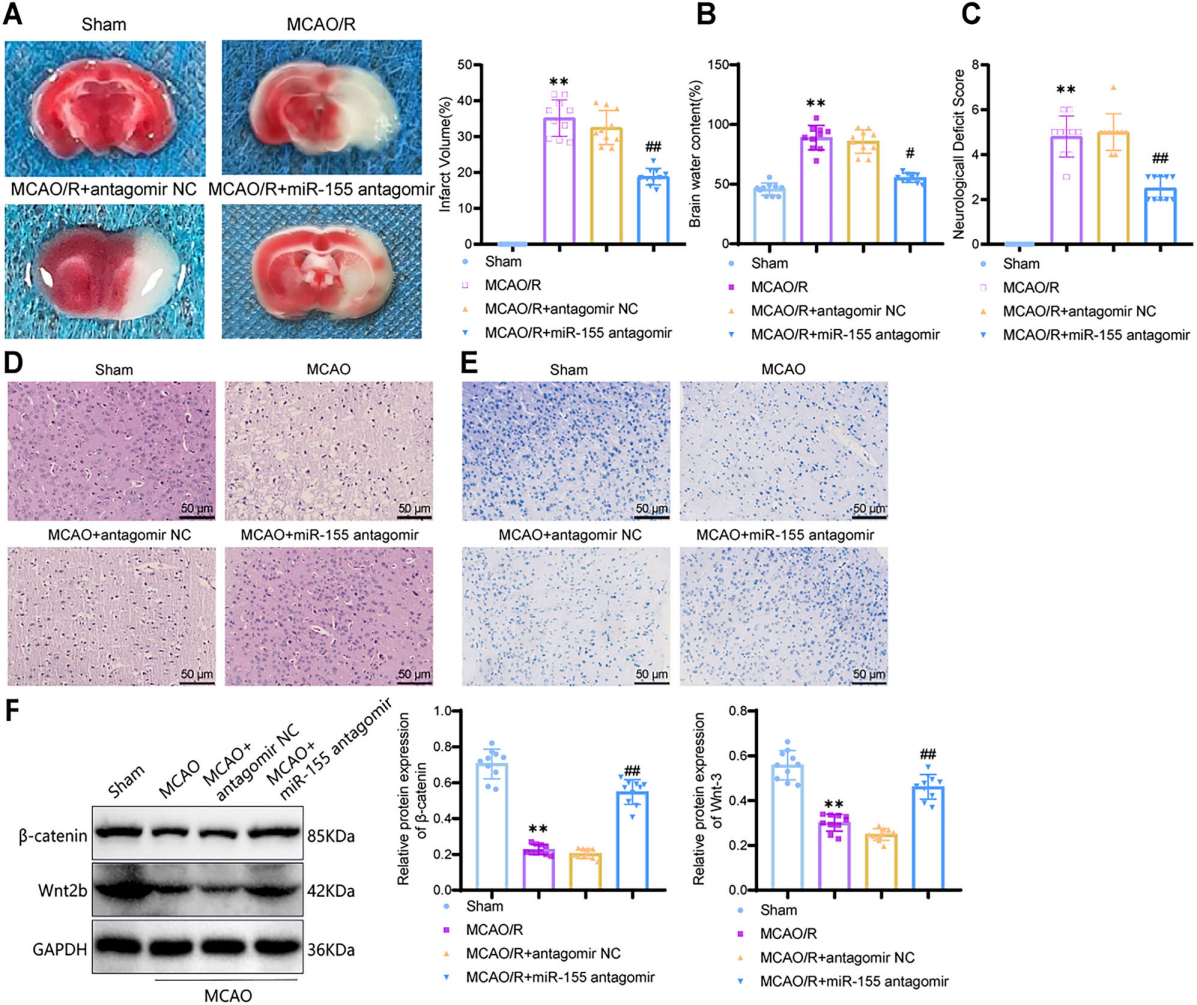
Effects of miR-155 antagomir on cerebral infarction and neural damage in MCAO mice. ***A***, TTC staining used to detect cerebral infarction in the ipsilateral hemisphere of mice in each group and measurement of infarct volume; ***B***, Determination of brain water content in the ipsilateral hemisphere; ***C***, Evaluation of neural functional impairment; ***D***, H&E staining of the hippocampal tissue; scale bar, 50 μm; ***E***, Nissl staining of the hippocampal tissue; scale bar, 50 μm; ***F***, Western blot analysis of protein expression levels of β-catenin and Wnt-3. ** indicates a *p* value of <0.01 compared with the Sham group; # indicates a *p* value of <0.05; ## indicates a *p* value of <0.01 compared with the MCAO + antagomir NC group; *n* = 10.

Subsequently, we conducted TTC staining and infarct volume measurements (Fig. 3*A*). The results showed that, compared with the Sham group, the infarct volume in the MCAO group was significantly increased (*p* < 0.0001; 95% CI, −39.57 to −30.75). However, pretreatment with miR-155 antagomir significantly reduced the infarct volume in MCAO mice (*p* < 0.0001; 95% CI, 9.264–18.08). Subsequently, we assessed the degree of brain edema by measuring brain water content (Fig. 3*B*). The results indicated that, compared with the Sham group, brain edema (brain water content) was significantly increased in the MCAO group (*p* < 0.0001; 95% CI, −59.79 to −31.71), while brain water content was significantly reduced in mice pretreated with miR-155 antagomir (*p* < 0.0001; 95% CI, 19.67–47.75). No infarction or edema formation was observed in the Sham group, indicating that miR-155 antagomir provides a certain degree of protection against cerebral infarction and brain edema in mice.

10.1523/ENEURO.0347-24.2024.f3-1 Figure 3-1 **Construction of MCAO mouse model and detection of miR-155 expression levels.** Note: (A) Pre-treatment of mice with miR-155 antagomir or antagomir NC via brain injection, followed by construction of MCAO model; (B) Detection of miR-155 expression levels in each group using qRT-PCR. ** indicates significant difference compared to the Sham group (*P* *<* 0.01); ## indicates significant difference compared to the MCAO + antagomir NC group (*P* *<* 0.01); n = 10. Download Figure 3-1, TIF file.

Neurological functional deficits were assessed using the classic Zea–Longa scoring method (Fig. 3*C*). MCAO mice showed evident neurological functional impairment compared with the Sham group (*p* < 0.0001; 95% CI, −5.605 to −3.995), while mice in the MCAO + miR-155 antagomir group demonstrated some recovery of neurological function (*p* < 0.0001; 95% CI, 1.695–3.305). Ischemic brain injury typically leads to nerve damage and morphological changes (Arai et al., 2001). Therefore, H&E staining was used to evaluate neuronal damage in MCAO mice (Fig. 3*D*). The H&E staining results showed that neurons in the Sham group exhibited intact morphology, clear structure, and prominent nucleoli without pathological alterations. In the MCAO group, neurons in the infarct core region appeared disordered, sparse, and swollen, with nuclear destruction and a significant reduction in surviving neurons. Treatment with miR-155 antagomir in MCAO mice that significantly attenuated neuronal damage maintained normal neuronal structure and increased the number of neurons. Nissl staining was performed to assess neuronal morphology further (Fig. 3*E*). Neurons in the Sham group had normal morphology, with clear cell contours, dense structure, and visible nucleoli. In the MCAO group, some neurons showed enlarged perineuronal spaces, swelling or shrinkage, the disappearance of nucleoli, and nuclear membrane dissolution. In comparison with the MCAO + antagomir NC group, MCAO + miR-155 antagomir mice exhibited partial relief of neuronal damage, with fewer degenerating cells, reduced cellular deformation, and increased numbers of viable cells. These results indicate that miR-155 antagomir can alleviate ischemic brain injury and neuronal damage.

Confirmation of the targeting relationship between miR-155 and Wnt2b was obtained through dual-luciferase reporter gene assays. The Wnt/β-catenin pathway, a highly conserved pathway, primarily plays a role in regulating neuronal survival and cell apoptosis in ischemic brain injury (Zhan et al., 2019). To investigate whether miR-155 antagomir regulates the Wnt/β-catenin signaling pathway in ischemic brain injury, we measured the expression levels of Wnt/β-catenin pathway–associated proteins using Western blot analysis (Fig. 3*F*). Compared with the Sham group, MCAO mice exhibited a significant decrease in the expression levels of Wnt/β-catenin pathway–related proteins (β-catenin, *p* < 0.0001; 95% CI, 0.4100–0.5480; Wnt3, *p* < 0.0001; 95% CI, 0.1988–0.3154), which were significantly increased after miR-155 antagomir treatment (β-catenin, *p* < 0.0001; 95% CI, −0.4139 to −0.2759; Wnt3, *p* < 0.0001; 95% CI, −0.2714 to −0.1548).

Overall, these results demonstrate that miR-155 antagomir can regulate the Wnt/β-catenin signaling pathway and may impact ischemic brain injury.

### Protective effects of miR-155 antagomir on inflammation and BBB in ischemic brain injury

**Figure 4.**
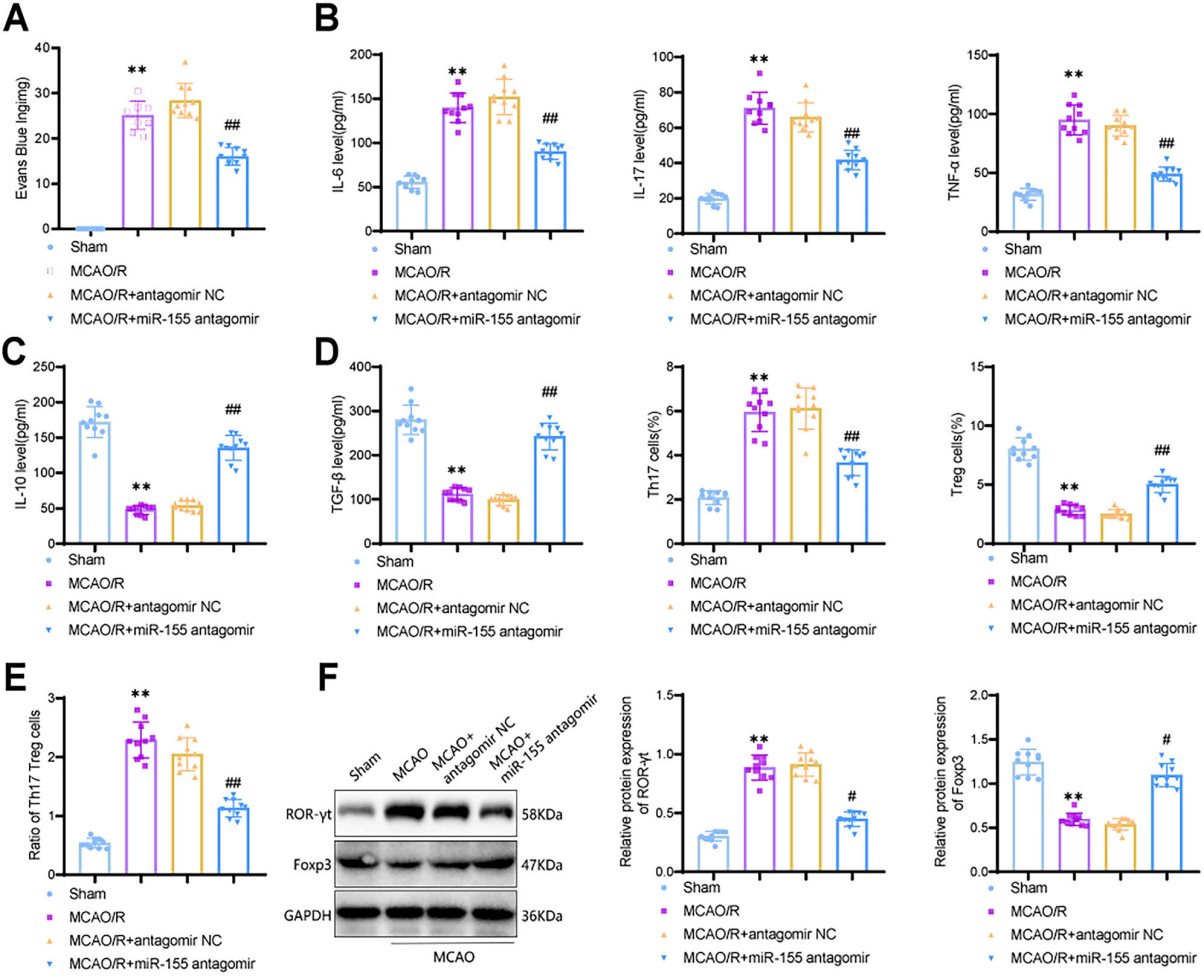
Reduction of Th17/Treg cell ratio in MCAO mice by miR-155 antagomir. ***A***, Evans blue extravasation indicating the extent of BBB disruption; ***B***, Serum levels of proinflammatory cytokines IL-6, IL-17, and TNF-α measured by ELISA; ***C***, Serum levels of anti-inflammatory cytokines IL-10 and TGF-β measured by ELISA; ***D***, Flow cytometry analysis and determination of Th17 and Treg cell proportions; ***E***, Th17/Treg cell ratio; ***F***, Protein expression levels of Foxp3 and ROR-γt detected by Western blotting. ** indicates a *p* value of <0.01 compared with the Sham group; # indicates a *p* value of <0.05; ## indicates a *p* value of <0.01 compared with the MCAO + antagomir NC group; *n* = 10.

The disruption of the BBB promotes injury and increases the risk of bleeding (Jiang et al., 2018). The extent of BBB leakage was measured using Evans blue dye (Fig. 4*A*). The results showed that, compared with the Sham group, mice subjected to MCAO surgery exhibited a significant increase in BBB leakage (*p* < 0.0001; 95% CI, −31.80 to −20.10), whereas treatment with miR-155 antagomir significantly improved BBB integrity (*p* < 0.0001; 95% CI, 7.861–19.56). One of the causes of BBB disruption is the occurrence of inflammation after disease (Shichita et al., 2012). Therefore, we assessed the expression levels of proinflammatory cytokines, including IL-6, IL-17, and TNF-α (Fig. 4*B*), as well as anti-inflammatory cytokines, such as IL-10 and TGF-β (Fig. 4*C*). The results indicated that the levels of proinflammatory cytokines were significantly increased (IL-6, *p* < 0.0001; 95% CI, −101.4 to −67.44; IL-17, *p* < 0.0001; 95% CI, −59.55 to −42.95; TNF-α, *p* < 0.0001; 95% CI, −73.72 to −52.80), and the levels of anti-inflammatory cytokines were significantly decreased in mice after MCAO surgery (IL-10, *p* < 0.0001; 95% CI, 105.8–141.6; TGF-β, *p* < 0.0001; 95% CI, 139.1–197.6). However, treatment with miR-155 antagomir effectively reversed these results, suggesting its ability to reduce inflammation and improve ischemic brain injury (IL-6, *p* < 0.0001; 95% CI, 44.93–78.93; IL-17, *p* < 0.0001; 95% CI, 15.87–32.47; TNF-α, *p* < 0.0001; 95% CI, 30.69–51.60; IL-10, *p* < 0.0001; 95% CI, −99.27 to −63.47; TGF-β, *p* < 0.0001; 95% CI, −172.6 to −114.1).

10.1523/ENEURO.0347-24.2024.f4-1 Figure 4-1 **Analysis of changes in Th17 and Treg cells by flow cytometry.** Note: Flow cytometry was used to detect the frequency of Th17 and Treg cells in peripheral blood. In the flow cytometry plot of Th17 cells, the x-axis represents FITC/CD4, and the y-axis represents PE/IL-17. In the flow cytometry plot of Treg cells, the x-axis represents FITC/CD4, and the y-axis represents PE/Foxp3. Download Figure 4-1, TIF file.

The inflammatory cascade reaction in ischemic stroke can lead to changes in the immune system. Treg and Th17 cells are distinct subsets of CD4^+^ T-lymphocytes that antagonize each other and maintain relative immune balance (Lee, 2018). Treg cells exert immunosuppressive and anti-inflammatory effects by secreting anti-inflammatory cytokines, such as IL-10 and TGF-β, while Th17 cells, regulated by the nuclear transcription factor ROR-γt, specifically secrete IL-17 and are induced to differentiate by IL-6 and TGF-β (Yang et al., 2008). A key characteristic of Treg cells is the expression of Foxp3 protein, which is crucial for maintaining Th17/Treg cell balance in inflammation and autoimmune diseases (J. Lu et al., 2018; Y. Lu et al., 2018). Therefore, we detected the quantities of Th17 and Treg cells using flow cytometry, with the results displayed in the Q2 quadrant (Extended Data Fig. 4-1) and quantified the ratio of Th17 to Treg cells (Fig. 4*D*). The results showed an imbalance in the Th17/Treg cell ratio induced by ischemic brain injury (Fig. 4*E*), characterized by a higher proportion of Th17 cells and a lower proportion of Treg cells. However, treatment with miR-155 antagomir effectively restored the balance of the Th17/Treg cell ratio. Additionally, to further examine whether there were changes in the protein expression levels of ROR-γt and Foxp3, we performed Western blot analysis (Fig. 4*F*). The results demonstrated that, compared with the Sham group, the MCAO group exhibited a decrease in Foxp3 expression (*p* < 0.0001; 95% CI, 0.5152–0.7788) and an increase in ROR-γt expression (*p* < 0.0001; 95% CI, −0.6811 to −0.4851). Treatment with miR-155 antagomir significantly reversed the protein expression levels of ROR-γt and Foxp3 (Foxp3, *p* < 0.0001; 95% CI, −0.6888 to −0.4252; ROR-γt, *p* < 0.0001; 95% CI, 0.3660–0.5620).

Overall, the findings indicate that miR-155 antagomir can effectively alleviate inflammation, reduce BBB leakage, and promote the balance of the Th17/Treg cell ratio, thereby improving ischemic brain injury.

### miR-155 inhibitor improves ischemic brain injury by activating the Wnt/β-catenin signaling pathway and modulating Th17/Treg cell balance

**Figure 5.**
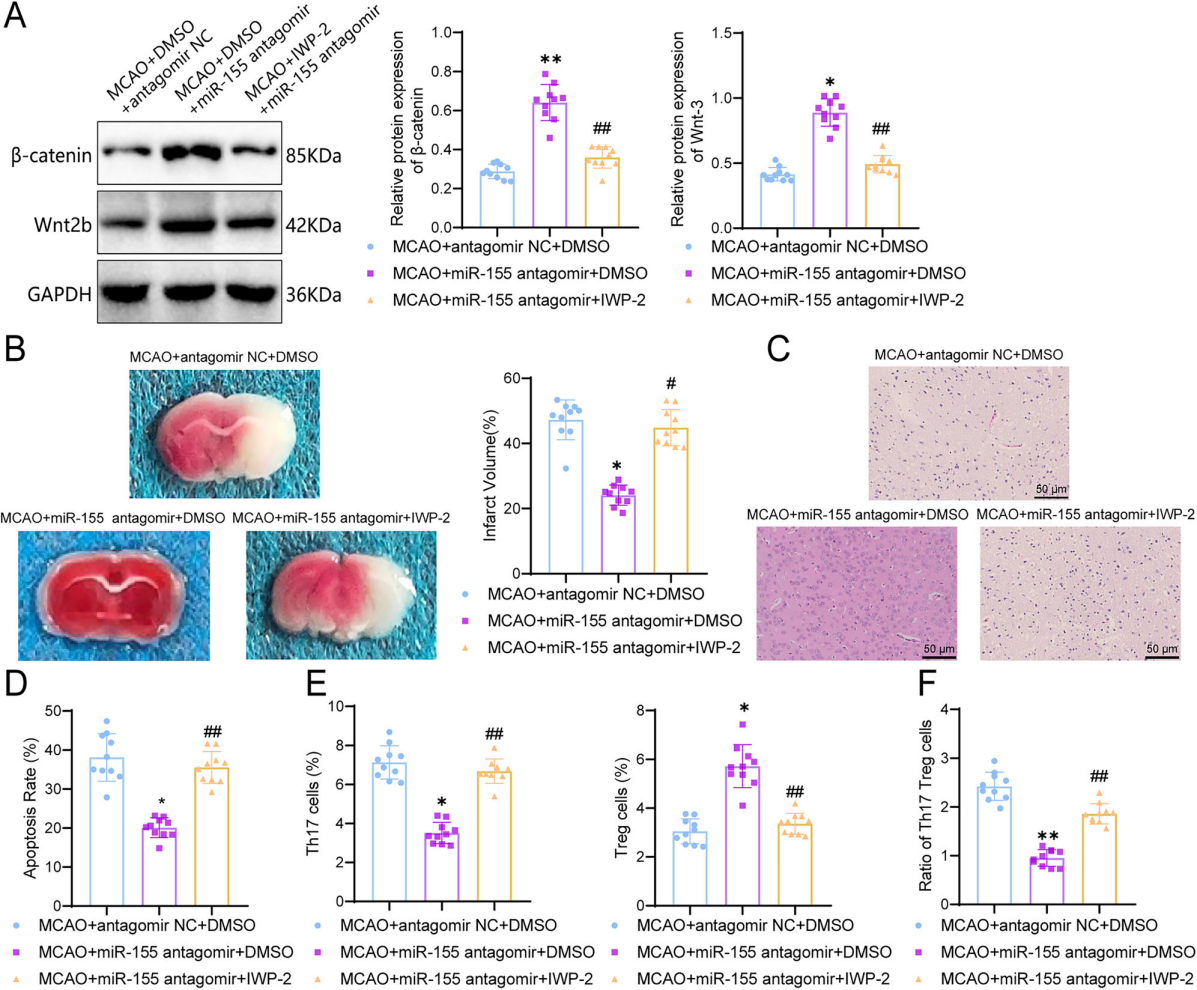
The effect of inhibiting the Wnt/β-catenin pathway on cerebral infarction and neuronal damage in MCAO mice. ***A***, Protein expression of β-catenin and Wnt2b detected using Western blot; ***B***, Evaluation of cerebral infarction in the ipsilateral hemisphere using TTC staining and measurement of infarct volume; ***C***, H&E stain of hippocampal tissues with a scale bar of 50 μm; ***D***, Evaluation of cell apoptosis using TUNEL assay, followed by quantitative analysis; ***E***, Measurement and analysis of Th17 and Treg cell ratio using flow cytometry; ***F***, Measurement and analysis of Th17/Treg cell ratio using flow cytometry. *p < 0.05; **p < 0.01 indicates significant difference compared with the MCAO + antagomir NC + DMSO group; ^#^*p *< 0.05; ^##^*p *< 0.01 indicates significant difference compared with the MCAO + miR-155 antagomir + DMSO group; *n* = 10.

Recent studies have found a connection between the Wnt/β-catenin signaling pathway and the differentiation of Th17 cells, which plays an important role in CD4^+^ T-cell–mediated inflammation (Dai et al., 2016). Therefore, in order to investigate whether miR-155 regulates the Wnt/β-catenin signaling pathway to influence the Th17/Treg cell balance and improve ischemic brain injury, we treated MCAO mice with the Wnt/β-catenin signaling pathway inhibitor IWP-2 and performed grouping experiments with the addition of miR-155 antagomir. Western Blot analysis of Wnt/β-catenin protein expression revealed that compared with the MCAO + antagomir NC + DMSO group, the MCAO + miR-155 antagomir + DMSO group showed a significant increase in Wnt/β-catenin signaling pathway-related protein expression (β-catenin, *p* < 0.0001; 95% CI, −0.4260 to −0.2798; Wnt, *p* < 0.0001; 95% CI, −0.5613 to −0.3885; Fig. 5*A*). However, when miR-155 antagomir treatment was combined with IWP-2, the expression levels of Wnt/β-catenin signaling pathway-related proteins significantly decreased compared with the MCAO + miR-155 antagomir + DMSO group (*p* < 0.0001; 95% CI, 0.2087–0.3549; *p* < 0.0001, 95% CI, 0.3108–0.4836). These results indicate that IWP-2 can inhibit the Wnt/β-catenin signaling pathway, and miR-155 antagomir can effectively decrease this inhibitory effect.

10.1523/ENEURO.0347-24.2024.f5-1 Figure 5-1 **Analysis of changes in Th17 and Treg cells by flow cytometry.** Note: Flow cytometry was used to detect the frequency of Th17 and Treg cells in peripheral blood. In the flow cytometry plot of Th17 cells, the x-axis represents FITC/CD4, and the y-axis represents PE/IL-17. In the flow cytometry plot of Treg cells, the x-axis represents FITC/CD4, and the y-axis represents PE/Foxp3. Download Figure, TIF file.

To evaluate the extent of cerebral infarction in the different treatment groups of mice, TTC staining results were obtained (Fig. 5*B*). It was observed that miR-155 antagomir treatment effectively reduced the volume of cerebral infarction compared with the MCAO + antagomir NC + DMSO group (*p* < 0.0001; 95% CI, 17.52–28.80). However, when IWP-2 treatment was added to miR-155 antagomir pretreated MCAO mice, the area of cerebral infarction was increased compared with the mice that were only pretreated with miR-155 antagomir (*p* < 0.0001; 95% CI, −26.39 to −15.11). Subsequently, the pathological changes in the ischemic brain tissue were assessed using H&E staining (Fig. 5*C*). The samples in the MCAO + antagomir NC + DMSO group showed fewer positive cell nuclei and a significant amount of disarray between cells, which was alleviated in the miR-155 antagomir pretreatment group. In contrast, the hippocampal tissue damage in the MCAO + miR-155 antagomir + IWP-2 group was significantly aggravated. The TUNEL method was used to evaluate neuronal apoptosis (Fig. 5*D*). Neuronal apoptosis was characterized by a significant increase in TUNEL-positive cells in the MCAO + antagomir NC + DMSO mice (*p* < 0.0001; 95% CI, 13.08–23.00), while the MCAO + miR-155 antagomir + DMSO group showed significantly lower neuronal apoptosis compared with the MCAO + miR-155 antagomir + IWP-2 group (*p* < 0.0001; 95% CI, −20.41 to −10.49). miR-155 antagomir can effectively inhibit the activation of the Wnt/β-catenin signaling pathway and alleviate neuronal apoptosis.

To investigate whether the Th17/Treg cell ratio was affected, flow cytometry was used to measure the quantity of Th17 and Treg cells. The flow cytometry results were displayed in the Q2 area (Extended Data Fig. 5-1), and the quantity of Th17 and Treg cells was quantified (Fig. 5*E*). The results demonstrated a significant increase in the Th17/Treg cell ratio after inhibiting the Wnt/β-catenin signaling pathway using IWP-2 compared with the MCAO + miR-155 antagomir + DMSO group (Fig. 5*F*), indicating an increased imbalance. However, the miR-155 antagomir pretreatment group exhibited improved Th17/Treg cell ratio balance compared with the MCAO + antagomir NC + DMSO group.

In conclusion, miR-155 antagomir can activate the Wnt/β-catenin signaling pathway, thereby influencing Th17/Treg cell balance and improving ischemic brain injury.

### Inhibition of miR-155 improves OGD/R-induced cell damage and inflammation by activating the Wnt/β-catenin signaling pathway

**Figure 6.**
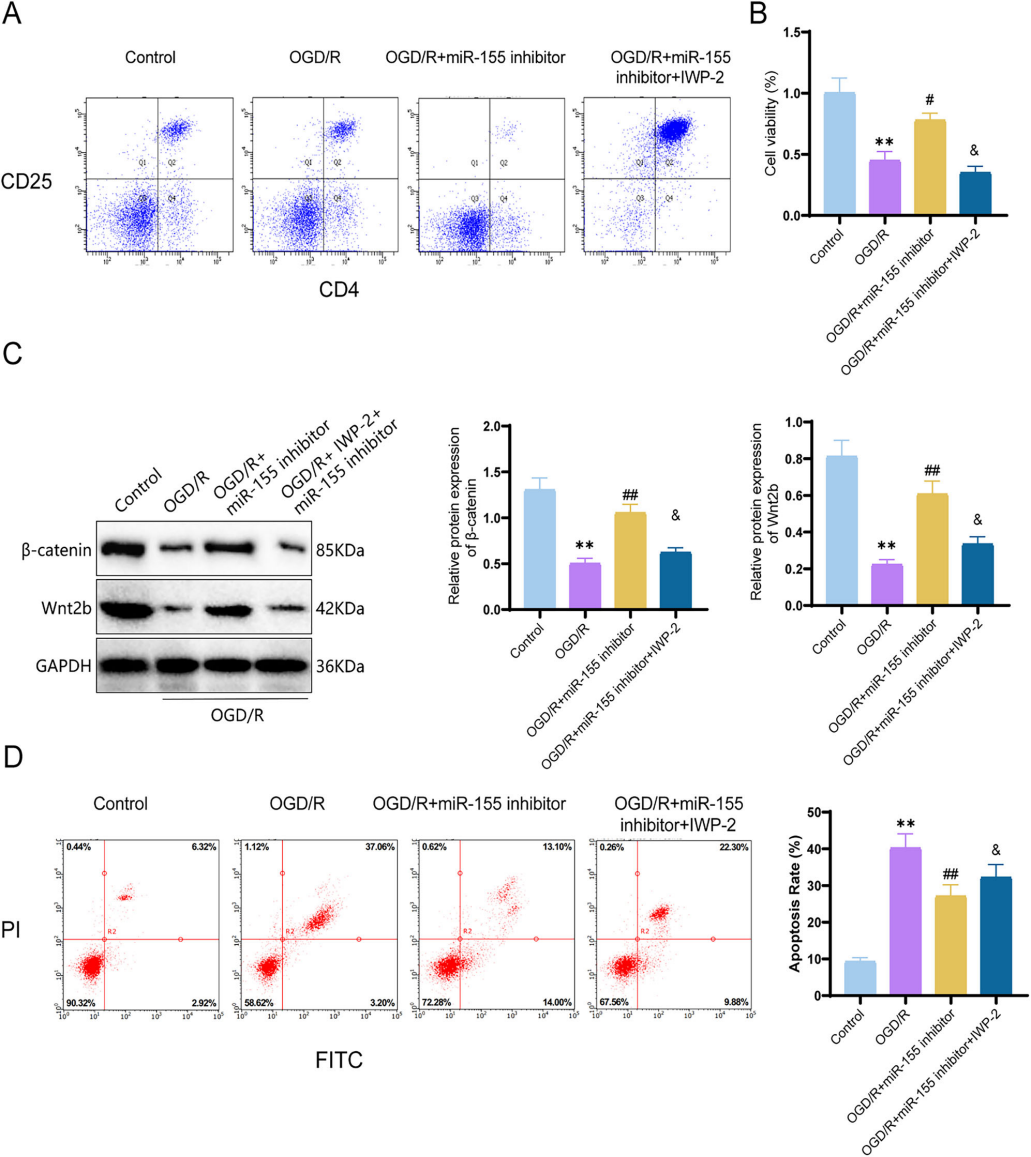
The effect of miR-155 inhibitor on OGD/R-induced neuronal apoptosis. ***A***, Representative flow cytometry histograms showing CD25 and Foxp3 expression in negatively selected splenocytes and CD4^+^ T-cells; ***B***, Cell viability assessed by CCK-8 assay; ***C***, Protein expression of β-catenin and Wnt2b detected using Western blot; ***D***, Flow cytometry analysis of cell apoptosis under different treatments. **p < 0.01 indicates significant difference compared with the control group; ^#^*p *< 0.05; ^##^*p *< 0.01 indicates significant difference compared with the OGD/R group; ^&^*p *< 0.05 indicates significant difference compared with the OGD/R + miR-155 inhibitor group. The cell experiments were repeated three times.

In our in vivo experiments, we have demonstrated that inhibition of miR-155 can activate the Wnt/β-catenin signaling pathway, thereby improving ischemic brain injury and neuronal apoptosis. Therefore, in order to investigate whether the inhibition of miR-155 affects the quantity of Treg cells and improves OGD/R-induced cell damage and inflammation by regulating the Wnt/β-catenin signaling pathway, we utilized a coculture method with Treg cells and neurons. Initially, we isolated primary cortical neurons from mice and constructed an OGD/R injury model in vitro. We performed identification of the cultured primary cortical neurons’ morphology and purity (Extended Data Fig. 6-1). After 2 d of cultivation, neurons exhibited a smaller size, relatively round cell bodies, and shorter neurites, while on the seventh day, they extended and formed a network. Immunofluorescence staining results showed that the cell bodies and neuronal processes expressed β-III tubulin, with a purity of over 90%, confirming successful isolation. Next, Treg cells were isolated from donor mice, and the enrichment rate of CD4 CD25 Treg cells was assessed using flow cytometry, which exceeded 95%, indicating successful isolation. The expression of the immune phenotypic marker Foxp3 in Treg cells was 82% (Fig. 6*A*). After a 48 h coculture period, cell viability was assessed in different treatment groups (Fig. 6*B*), with the OGD/R-induced group displaying the lowest cell viability (*p* = 0.0001; 95% CI, 0.3440–0.7626). However, cell viability increased upon downregulation of miR-155 expression (*p* = 0.0044; 95% CI, −0.5393 to −0.1207).

10.1523/ENEURO.0347-24.2024.f6-1 Figure 6-1 **Morphological observation and identification of primary cortical neurons.** Note: (A) Morphological image of neurons cultured for 2 days under an optical microscope; (B) Morphological image of neurons cultured for 7 days under an optical microscope; (C) Immunofluorescence staining for the expression of β-III Tubulin. β-III Tubulin is shown in red fluorescence, and the cell nucleus is marked with blue fluorescence. The scale bar is 50 μm. The cell experiment was repeated three times. Download Figure 6-1, TIF file.

Subsequently, we evaluated the expression levels of proteins related to the Wnt/β-catenin signaling pathway. Western blot results (Fig. 6*C*) showed that compared with the control group, the protein expression of β-catenin and Wnt2b in cells subjected to OGD/R induction significantly decreased (β-catenin, *p* < 0.0001; 95% CI, 0.5747–1.036; Wnt2b, *p* < 0.0001; 95% CI, 0.4275–0.7485), while their expression levels increased after miR-155 knockdown (β-catenin, *p* = 0.0003; 95% CI, −0.7836 to −0.3224; Wnt2b, *p* = 0.0003; 95% CI, −0.5435 to −0.2225). Furthermore, after adding the Wnt/β-catenin pathway inhibitor, the protein expression levels of β-catenin and Wnt2b decreased compared with the OGD/R + miR-155 inhibitor group (*p* = 0.0003; 95% CI, 0.2014–0.6626; *p* = 0.0003; 95% CI, 0.1115–0.4325). These results indicate that the inhibition of miR-155 activates the Wnt/β-catenin signaling pathway in cells subjected to OGD/R induction. Cell apoptosis was assessed (Fig. 6*D*), and it was found that compared with the control group, OGD/R induction significantly increased cell apoptosis (*p* < 0.0001; 95% CI, −43.42 to −20.48). Conversely, in the OGD/R + miR-155 inhibitor group, cell apoptosis significantly decreased (*p* = 0.0035; 95% CI, 12.24–35.18). However, when the Wnt/β-catenin signaling pathway was inhibited in the OGD/R + miR-155 inhibitor group, cell apoptosis increased (*p* = 0.2564; 95% CI, −25.23 to −2.294).

These results confirm that miR-155 inhibition can improve OGD/R-induced cell damage by activating the Wnt/β-catenin signaling pathway, consistent with our animal experimental findings.

### miR-155 inhibitor improves neural injury and suppresses inflammatory response by modulating Treg cell population

**Figure 7.**
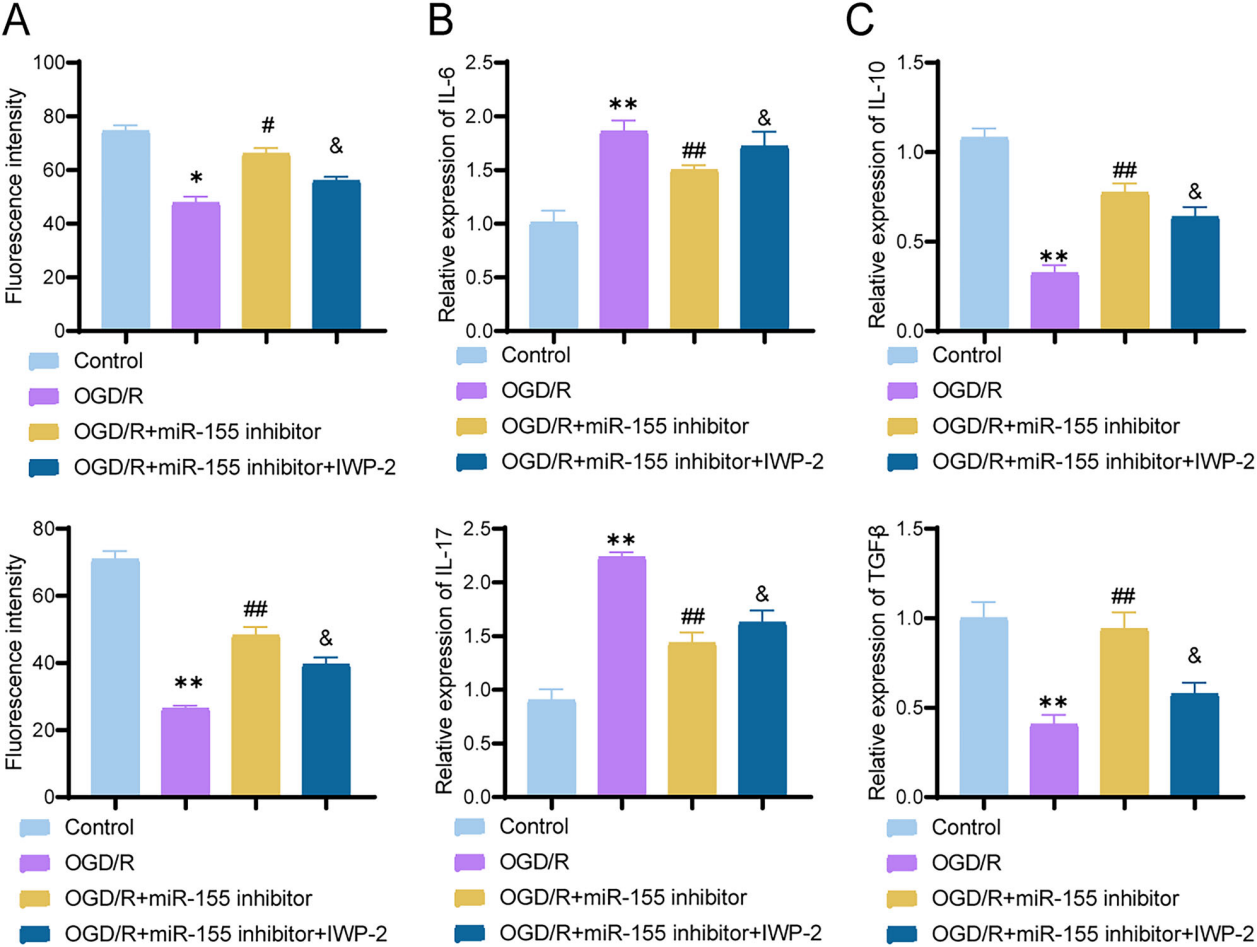
The effect of miR-155 inhibitor on OGD/R-induced neuronal inflammation. ***A***, Dual immunofluorescence staining showing the distribution of Foxp3 (red) and NeuN (green), with quantitative analysis; scale bar, 50 μm. ***B***, Quantitative detection of proinflammatory cytokines IL-6 and IL-17 expression using RT-qPCR; ***C***, Quantitative detection of anti-inflammatory cytokines IL-10 and TGF-β expression using RT-qPCR. ** indicates significant difference compared with the control group (*p* < 0.01); ## indicates significant difference compared with the OGD/R group (*p* < 0.01); & indicates significant difference compared with the OGD/R + miR-155 inhibitor group (*p* < 0.01). The cell experiments were repeated three times.

In our animal experiment, we observed that the miR-155 antagomir influenced the quantity of Treg cells and consequently impacted neural injury. To evaluate the potential effects of Treg cells on neurons, we conducted a dual immunofluorescence labeling of Foxp3 (Treg marker) and NeuN (neuronal marker; Fig. 7*A*). Compared with the control group, the OGD/R group showed a significant decrease in NeuN expression (*p* = 0.0001; 95% CI, 18.46–35.14), indicating that OGD/R induction affected the quantity and activity of neuronal cells. In addition, the Foxp3 expression was significantly lower in the OGD/R group compared with the control group (*p* = 0.0007; 95% CI, 35.81–52.99), whereas the OGD/R group treated with the miR-155 inhibitor showed a significant promotion of Foxp3 expression (*p* = 0.003; 95% CI, −26.64 to −9.962). However, in the OGD/R group treated with both the miR-155 inhibitor and IWP-2, the expression of Foxp3 decreased when compared with the OGD/R + miR-155 inhibitor group (*p* = 0.0274; 95% CI, 1.662–18.34). Thus, the miR-155 inhibitor was able to facilitate an increase in Treg cells, thereby alleviating neuronal damage caused by OGD/R.

Next, we examined the expression of inflammatory-related factors. qRT-PCR analysis demonstrated a significant increase in the expression levels of the proinflammatory cytokines IL-6 and IL-17 in neurons after OGD/R induction (IL-6, *p* < 0.0001; 95% CI, −1.121 to −0.5723; IL-17, *p* < 0.0001; 95% CI, −1.577 to −1.083; Fig. 7*B*). The miR-155 inhibitor significantly reduced the expression of proinflammatory cytokines induced by OGD/R (*p* = 0.0134; 95% CI, 0.08233–0.6310; *p* < 0.0001; 95% CI, 0.5532–1.047), while IWP-2 antagonized the effect of the miR-155 inhibitor. As for the expression levels of the anti-inflammatory cytokines IL-10 and TGF-β, we observed a significant decrease in their expression levels in neurons after OGD/R induction (IL-10, *p* < 0.0001; 95% CI, 0.6227–0.8906; TGF-β, *p* < 0.0001; 95% CI, 0.3906–0.7968; Fig. 7*C*). The miR-155 inhibitor significantly enhanced the expression levels of anti-inflammatory cytokines (IL-10, *p* < 0.0001; 95% CI, −0.5840 to −0.3160; TGF-β, *p* = 0.0001; 95% CI, −0.7358 to −0.3296). Compared with the OGD/R + miR-155 inhibitor group, the addition of the inhibitor IWP-2 resulted in a significant decrease in the expression levels of IL-10 and TGF-β (IL-10, *p* = 0.0457; 95% CI, 0.002703–0.2706; TGF-β, *p* = 0.002; 95% CI, 0.1592–0.5654), suggesting an antagonistic effect between IWP-2 and the miR-155 inhibitor. In OGD/R-induced neurons, the miR-155 inhibitor decreased the expression of proinflammatory cytokines and increased the expression of anti-inflammatory cytokines, consequently alleviating the neuronal inflammatory response.

## Discussion

**Figure 8.**
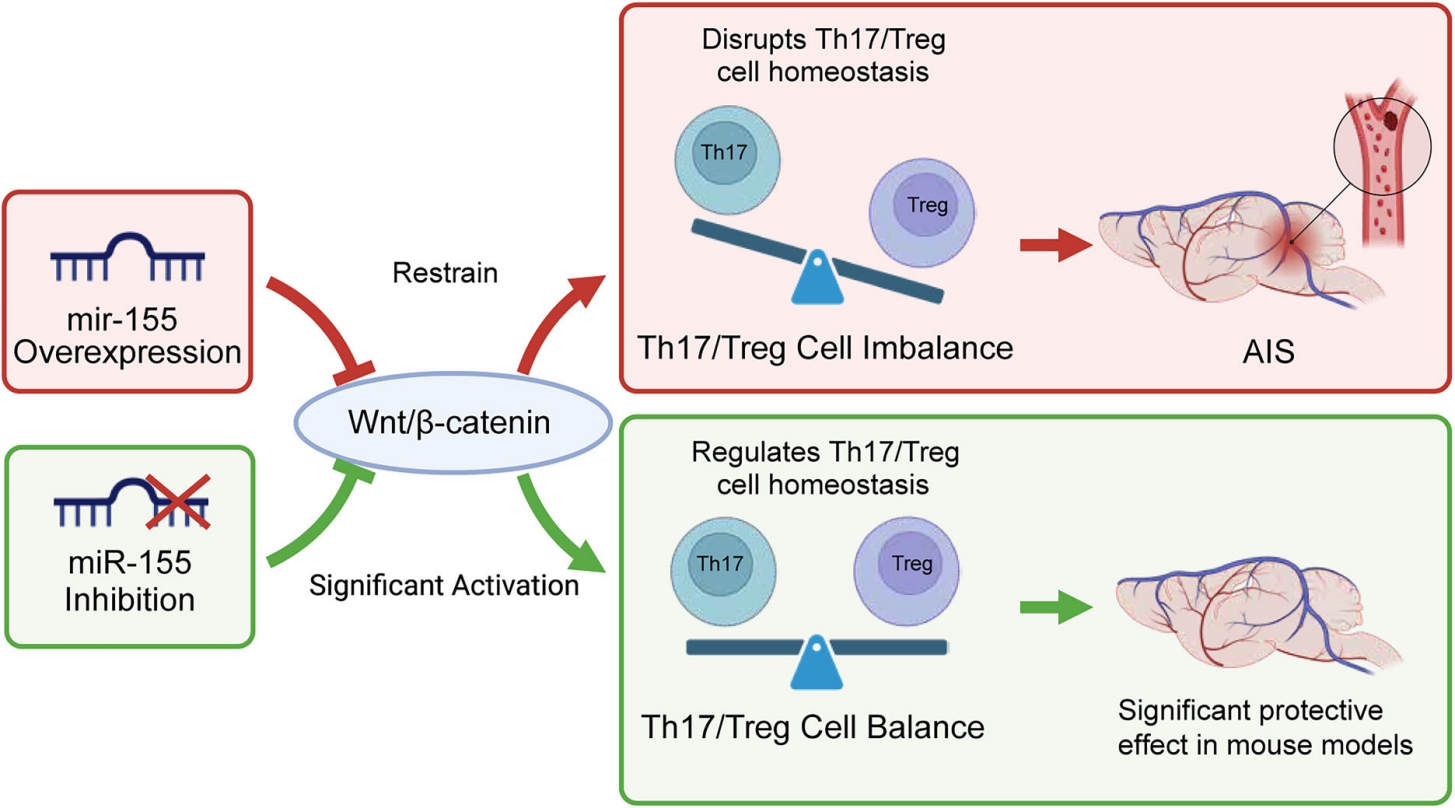
The modulation of the Wnt/β-catenin signaling pathway by miR-155 targeting affecting Th17/Treg cell balance in ischemic brain injury.

AIS is a severe neurological disorder closely associated with abnormal immune system activation and cell signaling pathway imbalance (Gong et al., 2021; Qiu et al., 2021; Cai et al., 2023). This study aims to investigate whether inhibiting miR-155 can improve the imbalance of Th17/Treg cells and activate the Wnt/β-catenin signaling pathway to provide protective effects against stroke (Fig. 8). Through cell experiments and a mouse model, we found that treatment with miR-155 antagomir significantly activates the Wnt/β-catenin signaling pathway and improves the ratio of Th17/Treg cells. These findings provide important evidence for further research on miR-155 as a potential therapeutic strategy for stroke (Zingale et al., 2021; Ruan et al., 2023; Zhang et al., 2023).

miR-155 is an important miRNA that plays a crucial regulatory role in the immune system and inflammatory processes (Cao et al., 2022; Ji et al., 2022; C. Wang et al., 2023; S. Wang et al., 2023). Previous studies have shown that miR-155 is closely related to the occurrence and development of inflammatory diseases and cancer (Bu et al., 2021; Wen et al., 2021; Ginckels and Holvoet, 2022). In stroke, the overexpression of miR-155 has been reported and is associated with the imbalance of Th17/Treg cells (Li et al., 2022; Jiang et al., 2023).

In cancer-related research, the transcription of miR-155 is regulated by nuclear factor κB (NF-κB), with p300 enhancing NF-κB-dependent expression of miR-155. Overexpression of miR-155 significantly inhibits hepatocyte apoptosis and promotes cell proliferation, while inhibiting miR-155 induces G(0)/G(1) cell cycle arrest. Upregulation of miR-155 leads to the accumulation of β-catenin in the nucleus, accompanied by increases in cyclin D1, c-myc, and survivin. Gain- and loss-of-function studies indicate that miR-155 promotes hepatocyte proliferation and tumorigenesis by enhancing Wnt signaling in vitro and in vivo, and DKK1 (a Wnt pathway inhibitor) overexpression suppresses the biological effects of miR-155 in hepatocytes (Zhang et al., 2012). In AIS, however, miR-155 plays an opposite role; inhibition of miR-155 in poststroke mice significantly alters the timeline of expression for key cytokines and inflammation-related molecules, thus affecting inflammation and tissue repair following experimental cerebral ischemia (Pena-Philippides et al., 2016).

Compared with previous studies, our research findings are consistent with the existing literature and provide further insight into the regulation of Wnt/β-catenin signaling activation and Th17/Treg cell balance by miR-155 inhibition (Shi et al., 2021; Huang et al., 2022). Additionally, our study addresses the limitations of previous research and offers new evidence supporting the role of miR-155 in stroke mechanisms.

The scientific significance of our study lies in the validation, at the cellular and animal model levels, of the protective effect of miR-155 inhibition in AIS. We have also revealed the mechanism by which it activates the Wnt/β-catenin signaling pathway to improve Th17/Treg cell imbalance. This discovery provides a new perspective to further understand the pathogenesis of stroke and offers important evidence for the development of miR-155-targeted therapeutic strategies. Moreover, through bioinformatics analysis, we have identified potential target genes associated with the Wnt/β-catenin signaling pathway and Th17/Treg cell imbalance, thereby providing candidate genes for future investigations. Therefore, our study holds significant scientific value in advancing translational research for stroke treatment.

However, this study has certain limitations. Firstly, while animal models can provide preliminary evidence, the complex physiological and pathological environment in humans may lead to different responses. Therefore, further clinical studies are needed to verify the feasibility and effectiveness of these findings. Secondly, although this study identified potential target genes through high-throughput sequencing and bioinformatics analysis, the exact functions and interactions of these genes still require further exploration. Additionally, the relatively small sample size may introduce bias and limitations; for instance, only one intersecting gene was identified between the differentially expressed genes and miR-155 target genes, whereas there may be additional potential targets worth exploring. Finally, a limitation of our study is the lack of evaluation of the long-term effects of miR-155 antagomir treatment poststroke. Given that miR-155 antagomir promotes an increase in Treg cells, it is plausible that this treatment could slow the progression of chronic neuroinflammation. Future investigations should include long-term follow–up studies to provide a more comprehensive assessment of its impact on chronic neuroinflammatory processes.

In terms of future directions, further research could delve into the relationship between miR-155 and the mechanisms underlying stroke, exploring regulatory mechanisms of the Wnt/β-catenin signaling pathway and Th17/Treg cell imbalance in greater depth. It would also be valuable to investigate whether miR-155 may target key genes beyond the Wnt/β-catenin pathway that are relevant to AIS and could play roles in neuroprotection. Furthermore, other immune-related cells (such as microglia and astrocytes) may also have significant roles in stroke pathology, and future research plans to analyze these cell groups to provide a more comprehensive perspective on the immune response in AIS. Future studies could further examine miR-155’s regulatory effects within the Wnt/β-catenin pathway, for example, by creating Wnt2b knock-out mouse models to clarify the mechanisms of action of miR-155 antagomir. Combining pharmacological and gene therapy approaches could further validate miR-155’s therapeutic potential and design larger-scale clinical trials to evaluate its clinical feasibility and effectiveness. Given the complexity of stroke pathology, investigating gender differences or hormonal influences could also be an interesting direction, as stroke outcomes may vary between males and females. Through further research, we can deepen our understanding of the pathophysiological processes of stroke and offer more opportunities for developing new treatment strategies and interventions.

## Data Availability

All data can be provided as needed.

## Synthesis

Reviewing Editor: Eran Perlson, Tel Aviv University Decisions are customarily a result of the Reviewing Editor and the peer reviewers coming together and discussing their recommendations until a consensus is reached. When revisions are invited, a fact-based synthesis statement explaining their decision and outlining what is needed to prepare a revision will be listed below. The following reviewer(s) agreed to reveal their identity: NONE.

The authors answer the raised concerns

## References

[B1] Arai K, Ikegaya Y, Nakatani Y, Kudo I, Nishiyama N, Matsuki N (2001) Phospholipase A2 mediates ischemic injury in the hippocampus: a regional difference of neuronal vulnerability. Eur J Neurosci 13:2319–2323. 10.1046/j.0953-816x.2001.01623.x 11454037

[B2] Ayuk SM, Abrahamse H, Houreld NN (2016) The role of photobiomodulation on gene expression of cell adhesion molecules in diabetic wounded fibroblasts in vitro. J Photochem Photobiol B Biol 161:368–374. 10.1016/j.jphotobiol.2016.05.027 27295416

[B3] Basu A, Ramamoorthi G, Albert G, Gallen C, Beyer A, Snyder C, Koski G, Disis ML, Czerniecki BJ, Kodumudi K (2021) Differentiation and regulation of T^H^ cells: a balancing act for cancer immunotherapy. Front Immunol 12:669474. 10.3389/fimmu.2021.669474 34012451 PMC8126720

[B4] Borlongan MC, et al. (2021) IL-2/IL-2R antibody complex enhances Treg-induced neuroprotection by dampening TNF-α inflammation in an in vitro stroke model. Neuromolecular Med 23:540–548. 10.1007/s12017-021-08656-0 33830475 PMC8613084

[B5] Bu T, Li Z, Hou Y, Sun W, Zhang R, Zhao L, Wei M, Yang G, Yuan L (2021) Exosome-mediated delivery of inflammation-responsive Il-10 mRNA for controlled atherosclerosis treatment. Theranostics 11:9988–10000. 10.7150/thno.64229 34815799 PMC8581418

[B6] Cai W, et al. (2023) FOXP3+ macrophage represses acute ischemic stroke-induced neural inflammation. Autophagy 19:1144–1163. 10.1080/15548627.2022.2116833 36170234 PMC10012925

[B7] Cao J, Huo P, Cui K, Wei H, Cao J, Wang J, Liu Q, Lei X, Zhang S (2022) Follicular fluid-derived exosomal miR-143-3p/miR-155-5p regulate follicular dysplasia by modulating glycolysis in granulosa cells in polycystic ovary syndrome. Cell Commun Signal 20:61. 10.1186/s12964-022-00876-6 35534864 PMC9082924

[B8] Chen H, et al. (2016) IL-10 promotes neurite outgrowth and synapse formation in cultured cortical neurons after the oxygen-glucose deprivation via JAK1/STAT3 pathway. Sci Rep 6:30459. 10.1038/srep30459 27456198 PMC4960594

[B9] Chen Y, Li L, Zhang J, Cui H, Wang J, Wang C, Shi M, Fan H (2021) Dexmedetomidine alleviates lipopolysaccharide-induced hippocampal neuronal apoptosis via inhibiting the p38 MAPK/c-Myc/CLIC4 signaling pathway in rats. Mol Neurobiol 58:5533–5547. 10.1007/s12035-021-02512-9 34363182

[B10] Choi BR, Cave C, Na CH, Sockanathan S (2020) GDE2-dependent activation of canonical Wnt signaling in neurons regulates oligodendrocyte maturation. Cell Rep 31:107540. 10.1016/j.celrep.2020.107540 32375055 PMC7254694

[B11] Clarkson BD, et al. (2014) T cell-derived interleukin (IL)-21 promotes brain injury following stroke in mice. J Exp Med 211:595–604. 10.1084/jem.20131377 24616379 PMC3978271

[B12] Corada M, Nyqvist D, Orsenigo F, Caprini A, Giampietro C, Taketo MM, Iruela-Arispe ML, Adams RH, Dejana E (2010) The Wnt/beta-catenin pathway modulates vascular remodeling and specification by upregulating Dll4/Notch signaling. Dev Cell 18:938–949. 10.1016/j.devcel.2010.05.006 20627076 PMC8127076

[B13] Cyr P, Bronner SM, Crawford JJ (2016) Recent progress on nuclear receptor RORγ modulators. Bioorg Med Chem Lett 26:4387–4393. 10.1016/j.bmcl.2016.08.012 27542308

[B14] Dai W, Liu F, Li C, Lu Y, Lu X, Du S, Chen Y, Weng D, Chen J (2016) Blockade of Wnt/β-catenin pathway aggravated silica-induced lung inflammation through Tregs regulation on Th immune responses. Mediators Inflamm 2016:6235614. 10.1155/2016/6235614 27069316 PMC4812397

[B15] Daneman R, Agalliu D, Zhou L, Kuhnert F, Kuo CJ, Barres BA (2009) Wnt/beta-catenin signaling is required for CNS, but not non-CNS, angiogenesis. Proc Natl Acad Sci U S A 106:641–646. 10.1073/pnas.0805165106 19129494 PMC2626756

[B16] Fan L, Zhou L (2021) AG490 protects cerebral ischemia/reperfusion injury via inhibiting the JAK2/3 signaling pathway. Brain Behav 11:e01911. 10.1002/brb3.1911 33098244 PMC7821583

[B17] Ginckels P, Holvoet P (2022) Oxidative stress and inflammation in cardiovascular diseases and cancer: role of non-coding RNAs. Yale J Biol Med 95:129–152. 35370493 PMC8961704

[B18] Gong P, et al. (2021) The association of neutrophil to lymphocyte ratio, platelet to lymphocyte ratio, and lymphocyte to monocyte ratio with post-thrombolysis early neurological outcomes in patients with acute ischemic stroke. J Neuroinflammation 18:51. 10.1186/s12974-021-02090-6 33610168 PMC7896410

[B19] Greco R, Demartini C, Francavilla M, Zanaboni AM, Tassorelli C (2022) Antagonism of CGRP receptor: central and peripheral mechanisms and mediators in an animal model of chronic migraine. Cells 11:3092. 10.3390/cells11193092 36231054 PMC9562879

[B20] Hashemi M, et al. (2022) Long non-coding RNA (lncRNA) H19 in human cancer: from proliferation and metastasis to therapy. Pharmacol Res 184:106418. 10.1016/j.phrs.2022.106418 36038043

[B21] Huang HM, Wei YJ, Wang D, Wen XM (2022). Mechanism of miR-155 promoting drug resistance in childhood acute lymphoblastic leukemia by regulating Wnt/β-catenin signaling pathway. Zhongguo Shi Yan Xue Ye Xue Za Zhi 30:418–424. 10.19746/j.cnki.issn.1009-2137.2022.02.016 35395973

[B22] Huang S, Wang HL (2023) Salvianolic acid A improves nerve regeneration and repairs nerve defects in rats with brain injury by downregulating miR-212-3p-mediated SOX7. Kaohsiung J Med Sci 39:1222–1232. 10.1002/kjm2.12779 37987200 PMC11895910

[B23] Ji H, Kim TW, Lee WJ, Jeong SD, Cho YB, Kim HH (2022) Two circPPFIA1s negatively regulate liver metastasis of colon cancer via miR-155-5p/CDX1 and HuR/RAB36. Mol Cancer 21:197. 10.1186/s12943-022-01667-w 36224588 PMC9555114

[B24] Jiang Q, Su DY, Wang ZZ, Liu C, Sun YN, Cheng H, Li XM, Yan B (2021) Retina as a window to cerebral dysfunction following studies with circRNA signature during neurodegeneration. Theranostics 11:1814–1827. 10.7150/thno.51550 33408783 PMC7778582

[B25] Jiang X, Andjelkovic AV, Zhu L, Yang T, Bennett MVL, Chen J, Keep RF, Shi Y (2018) Blood-brain barrier dysfunction and recovery after ischemic stroke. Prog Neurobiol 163–164:144–171. 10.1016/j.pneurobio.2017.10.001 28987927 PMC5886838

[B26] Jiang Z, Shi L, Huang H, Lei D, Lou L, Jin Y, Sun J, Wang L (2023) Downregulated FTO promotes microRNA-155-mediated inflammatory response in cerebral ischemia/reperfusion injury. Neuroscience 526:305–313. 10.1016/j.neuroscience.2023.07.012 37437797

[B27] Jin W, et al. (2017) MUC1 induces acquired chemoresistance by upregulating ABCB1 in EGFR-dependent manner. Cell Death Dis 8:e2980. 10.1038/cddis.2017.378 28796259 PMC5596566

[B28] Lai Y, et al. (2020) Restoration of L-OPA1 alleviates acute ischemic stroke injury in rats via inhibiting neuronal apoptosis and preserving mitochondrial function. Redox Biol 34:101503. 10.1016/j.redox.2020.101503; (Retraction published Redox Biol. 2024 Sep;75:103271. doi: 10.1016/j.redox.2024.103271). 32199783 PMC7327985

[B29] Lee GR (2018) The balance of Th17 versus Treg cells in autoimmunity. Int J Mol Sci 19:730. 10.3390/ijms19030730 29510522 PMC5877591

[B30] Li B, Cao Y, Sun M, Feng H (2021) Expression, regulation, and function of exosome-derived miRNAs in cancer progression and therapy. FASEB J 35:e21916. 10.1096/fj.202100294RR 34510546

[B31] Li P, Luo X, Luo Z, He GL, Shen TT, Yu XT, Wang ZZ, Tan YL, Liu XQ, Yang XS (2022) Increased miR-155 in microglial exosomes following heat stress accelerates neuronal autophagy via their transfer into neurons. Front Cell Neurosci 16:865568. 10.3389/fncel.2022.865568 35634460 PMC9132214

[B32] Liang W, Lin C, Yuan L, Chen L, Guo P, Li P, Wang W, Zhang X (2019) Preactivation of Notch1 in remote ischemic preconditioning reduces cerebral ischemia-reperfusion injury through crosstalk with the NF-κB pathway. J Neuroinflammation 16:181. 10.1186/s12974-019-1570-9 31526384 PMC6747758

[B33] Liu C, et al. (2022) GLP-1R agonist exendin-4 protects against hemorrhagic transformation induced by rtPA after ischemic stroke via the Wnt/β-catenin signaling pathway. Mol Neurobiol 59:3649–3664. 10.1007/s12035-022-02811-9 35359227 PMC9148281

[B34] Liu J, et al. (2018) An integrated TCGA pan-cancer clinical data resource to drive high-quality survival outcome analytics. Cell 173:400–416.e11. 10.1016/j.cell.2018.02.052 29625055 PMC6066282

[B35] Lu H, Ashiqueali R, Lin CI, Walchale A, Clendaniel V, Matheson R, Fisher M, Lo EH, Selim M, Shehadah A (2023) Histone deacetylase 3 inhibition decreases cerebral edema and protects the blood-brain barrier after stroke. Mol Neurobiol 60:235–246. 10.1007/s12035-022-03083-z 36258136 PMC9758108

[B36] Lu J, Liu QH, Wang F, Tan JJ, Deng YQ, Peng XH, Liu X, Zhang B, Xu X, Li XP (2018) Exosomal miR-9 inhibits angiogenesis by targeting MDK and regulating PDK/AKT pathway in nasopharyngeal carcinoma. J Exp Clin Cancer Res 37:147. 10.1186/s13046-018-0814-3 30001734 PMC6044044

[B37] Lu Y, Kim NM, Jiang YW, Zhang H, Zheng D, Zhu FX, Liang R, Li B, Xu HX (2018) Cambogin suppresses dextran sulphate sodium-induced colitis by enhancing Treg cell stability and function. Br J Pharmacol 175:1085–1099. 10.1111/bph.14150 29352742 PMC5843713

[B38] Lu Z, Xiong Y, Feng X, Yang K, Gu H, Zhao X, Meng X, Wang Y (2023) Insulin resistance estimated by estimated glucose disposal rate predicts outcomes in acute ischemic stroke patients. Cardiovasc Diabetol 22:225. 10.1186/s12933-023-01925-1 37633905 PMC10464388

[B39] Luo A, et al. (2019) Exosome-derived miR-339-5p mediates radiosensitivity by targeting Cdc25A in locally advanced esophageal squamous cell carcinoma. Oncogene 38:4990–5006. 10.1038/s41388-019-0771-0 30858545

[B40] Ma P, Zhang Y, Chang L, Li X, Diao Y, Chang H, Hui L (2022) Tenecteplase versus alteplase for the treatment of patients with acute ischemic stroke: a systematic review and meta-analysis. J Neurol 269:5262–5271. 10.1007/s00415-022-11242-4 35776193

[B41] Moutabian H, et al. (2023) MicroRNA-155 and cancer metastasis: regulation of invasion, migration, and epithelial-to-mesenchymal transition. Pathol Res Pract 250:154789. 10.1016/j.prp.2023.154789 37741138

[B42] Pena-Philippides JC, Caballero-Garrido E, Lordkipanidze T, Roitbak T (2016) In vivo inhibition of miR-155 significantly alters post-stroke inflammatory response. J Neuroinflammation 13:287. 10.1186/s12974-016-0753-x 27829437 PMC5103429

[B43] Prossomariti A, Piazzi G, D'Angelo L, Miccoli S, Turchetti D, Alquati C, Montagna C, Bazzoli F, Ricciardiello L (2018) miR-155 is downregulated in familial adenomatous polyposis and modulates WNT signaling by targeting AXIN1 and TCF4. Mol Cancer Res 16:1965–1976. 10.1158/1541-7786.MCR-18-0115 30072583

[B44] Qiu X, Zhang M, Yang X, Hong N, Yu C (2013) *Faecalibacterium prausnitzii* upregulates regulatory T cells and anti-inflammatory cytokines in treating TNBS-induced colitis. J Crohns Colitis 7:e558–e568. 10.1016/j.crohns.2013.04.002 23643066

[B45] Qiu YM, Zhang CL, Chen AQ, Wang HL, Zhou YF, Li YN, Hu B (2021) Immune cells in the BBB disruption after acute ischemic stroke: targets for immune therapy? Front Immunol 12:678744. 10.3389/fimmu.2021.678744 34248961 PMC8260997

[B46] Ruan H, et al. (2023) Click chemistry extracellular vesicle/peptide/chemokine nanocarriers for treating central nervous system injuries. Acta Pharm Sin B 13:2202–2218. 10.1016/j.apsb.2022.06.007 37250158 PMC10213615

[B47] Salik B, et al. (2020) Targeting RSPO3-LGR4 signaling for leukemia stem cell eradication in acute myeloid leukemia. Cancer Cell 38:263–278.e6. 10.1016/j.ccell.2020.05.014 32559496

[B48] Scorziello A, Santillo M, Adornetto A, Dell'aversano C, Sirabella R, Damiano S, Canzoniero LM, Renzo GF, Annunziato L (2007) NO-induced neuroprotection in ischemic preconditioning stimulates mitochondrial Mn-SOD activity and expression via Ras/ERK1/2 pathway. J Neurochem 103:1472–1480. 10.1111/j.1471-4159.2007.04845.x 17680990

[B49] Shan Y, Wang L, Sun J, Chang S, Di W, Lv H (2023) Exercise preconditioning attenuates cerebral ischemia-induced neuronal apoptosis, Th17/Treg imbalance, and inflammation in rats by inhibiting the JAK2/STAT3 pathway. Brain Behav 13:e3030. 10.1002/brb3.3030 37143406 PMC10275560

[B50] Shi XY, Tao XF, Wang GW, He JF, Wu LF, Sun YZ, Sun XJ (2021) LncDBH-AS1 knockdown enhances proliferation of non-small cell lung cancer cells by activating the Wnt signaling pathway via the miR-155/AXIN1 axis. Eur Rev Med Pharmacol Sci 25:139–144. 10.26355/eurrev_202101_24377 33506901

[B51] Shichita T, Ago T, Kamouchi M, Kitazono T, Yoshimura A, Ooboshi H (2012) Novel therapeutic strategies targeting innate immune responses and early inflammation after stroke. J Neurochem 123:29–38. 10.1111/j.1471-4159.2012.07941.x 23050640

[B52] Sinha T, Panigrahi C, Das D, Chandra Panda A (2022) Circular RNA translation, a path to hidden proteome. Wiley Interdiscip Rev RNA 13:e1685. 10.1002/wrna.1685 34342387 PMC7613019

[B53] Stenman JM, Rajagopal J, Carroll TJ, Ishibashi M, McMahon J, McMahon AP (2008) Canonical Wnt signaling regulates organ-specific assembly and differentiation of CNS vasculature. Science 322:1247–1250. 10.1126/science.1164594 19023080

[B54] Strober W, Zhang F, Kitani A, Fuss I, Fichtner-Feigl S (2010) Proinflammatory cytokines underlying the inflammation of Crohn's disease. Curr Opin Gastroenterol 26:310–317. 10.1097/MOG.0b013e328339d099 20473158 PMC3681421

[B55] Sun X, Geng X, Zhang J, Zhao H, Liu Y (2015) miR-155 promotes the growth of osteosarcoma in a HBP1-dependent mechanism. Mol Cell Biochem 403:139–147. 10.1007/s11010-015-2344-z 25666090

[B56] Suofu Y, Wang X, He Y, Li F, Zhang Y, Carlisle DL, Friedlander RM (2020) Mir-155 knockout protects against ischemia/reperfusion-induced brain injury and hemorrhagic transformation. Neuroreport 31:235–239. 10.1097/WNR.0000000000001382 31876686

[B57] Ta S, et al. (2021) Variants of WNT7A and GPR124 are associated with hemorrhagic transformation following intravenous thrombolysis in ischemic stroke. CNS Neurosci Ther 27:71–81. 10.1111/cns.13457 32991049 PMC7804912

[B58] Tian DS, Qin C, Zhou LQ, Yang S, Chen M, Xiao J, Shang K, Bosco DB, Wu LJ, Wang W (2022) FSAP aggravated endothelial dysfunction and neurological deficits in acute ischemic stroke due to large vessel occlusion. Signal Transduct Target Ther 7:6. 10.1038/s41392-021-00802-1 34992208 PMC8738761

[B59] Wang C, Jiang J, Zhang X, Song L, Sun K, Xu R (2016) Inhibiting HMGB1 reduces cerebral ischemia reperfusion injury in diabetic mice. Inflammation 39:1862–1870. 10.1007/s10753-016-0418-z 27596007 PMC5112296

[B60] Wang C, et al. (2023) Nicotine exacerbates endothelial dysfunction and drives atherosclerosis via extracellular vesicle-miRNA. Cardiovasc Res 119:729–742. 10.1093/cvr/cvac140 36006370

[B61] Wang J, et al. (2022) The role of phosphatidylserine on the membrane in immunity and blood coagulation. Biomark Res 10:4. 10.1186/s40364-021-00346-0 35033201 PMC8760663

[B62] Wang S, Shi Y, Zhang Y, Yuan F, Mao M, Ma J (2023) Tregs depletion aggravates activation of astrocytes by modulating IL-10/GXP4 following cerebral infarction. Front Immunol 14:1255316. 10.3389/fimmu.2023.1255316 37622110 PMC10446222

[B63] Wang X, Sun H, Liao H, Wang C, Jiang C, Zhang Y, Cao Z (2017) MicroRNA-155-3p mediates TNF-α-inhibited cementoblast differentiation. J Dent Res 96:1430–1437. 10.1177/0022034517718790 28692806

[B64] Wen Q, Wang Y, Pan Q, Tian R, Zhang D, Qin G, Zhou J, Chen L (2021) MicroRNA-155-5p promotes neuroinflammation and central sensitization via inhibiting SIRT1 in a nitroglycerin-induced chronic migraine mouse model. J Neuroinflammation 18:287. 10.1186/s12974-021-02342-5 34893074 PMC8665643

[B65] Wu CS, Tsao DA, Chang HR (2021) Beta2-adrenergic receptor agonist inhibits keratinocyte proliferation by mechanisms involving nitric oxide. Postepy Dermatol Alergol 38:396–403. 10.5114/ada.2020.92918 34377119 PMC8330852

[B66] Wu H, Liu H, Zuo F, Zhang L (2018) Adenoviruses-mediated RNA interference targeting cytosolic phospholipase A2α attenuates focal ischemic brain damage in mice. Mol Med Rep 17:5601–5610. 10.3892/mmr.2018.8610 29484397 PMC5866000

[B67] Xiang B, Zhong P, Fang L, Wu X, Song Y, Yuan H (2019) miR-183 inhibits microglia activation and expression of inflammatory factors in rats with cerebral ischemia reperfusion via NF-κB signaling pathway. Exp Ther Med 18:2540–2546. 10.3892/etm.2019.7827 31572505 PMC6755485

[B68] Xie W, Zhu T, Dong X, Nan F, Meng X, Zhou P, Sun G, Sun X (2019) HMGB1-triggered inflammation inhibition of notoginseng leaf triterpenes against cerebral ischemia and reperfusion injury via MAPK and NF-κB signaling pathways. Biomolecules 9:512. 10.3390/biom9100512 31547018 PMC6843331

[B69] Xu LJ, Ouyang YB, Xiong X, Stary CM, Giffard RG (2015) Post-stroke treatment with miR-181 antagomir reduces injury and improves long-term behavioral recovery in mice after focal cerebral ischemia. Exp Neurol 264:1–7. 10.1016/j.expneurol.2014.11.007 25433215 PMC4324354

[B70] Xu Y, Zhang J, Gao F, Cheng W, Zhang Y, Wei C, Zhang S, Gao X (2023) Engeletin alleviates cerebral ischemia reperfusion-induced neuroinflammation via the HMGB1/TLR4/NF-κB network. J Cell Mol Med 27:1653–1663. 10.1111/jcmm.17758 37132060 PMC10273068

[B71] Xue X, Wang J, Fu K, Dai S, Wu R, Peng C, Li Y (2023) The role of miR-155 on liver diseases by modulating immunity, inflammation and tumorigenesis. Int Immunopharmacol 116:109775. 10.1016/j.intimp.2023.109775 36753984

[B72] Yang L, Anderson DE, Baecher-Allan C, Hastings WD, Bettelli E, Oukka M, Kuchroo VK, Hafler DA (2008) IL-21 and TGF-beta are required for differentiation of human T(H)17 cells. Nature 454:350–352. 10.1038/nature07021 18469800 PMC2760130

[B73] Yang QW, Lu FL, Zhou Y, Wang L, Zhong Q, Lin S, Xiang J, Li JC, Fang CQ, Wang JZ (2011) HMBG1 mediates ischemia-reperfusion injury by TRIF-adaptor independent Toll-like receptor 4 signaling. J Cereb Blood Flow Metab 31:593–605. 10.1038/jcbfm.2010.129 20700129 PMC3049514

[B74] Yao H, Zhang Y, Shu H, Xie B, Tao Y, Yuan Y, Shang Y, Yuan S, Zhang J (2019) Hyperforin promotes post-stroke neuroangiogenesis via astrocytic IL-6-mediated negative immune regulation in the ischemic brain. Front Cell Neurosci 13:201. 10.3389/fncel.2019.00201 31133816 PMC6514137

[B75] Zeng Y, Cao S, Yang H (2023) Circulating sex hormone-binding globulin levels and ischemic stroke risk: a Mendelian randomization study. Postgrad Med J 99:1272–1279. 10.1093/postmj/qgad083 37742091

[B76] Zhan L, Liu D, Wen H, Hu J, Pang T, Sun W, Xu E (2019) Hypoxic postconditioning activates the Wnt/β-catenin pathway and protects against transient global cerebral ischemia through Dkk1 inhibition and GSK-3β inactivation. FASEB J 33:9291–9307. 10.1096/fj.201802633R 31120770

[B77] Zhang JK, Li Y, Yu ZT, Jiang JW, Tang H, Tu GL, Xia Y (2023) OIP5-AS1 inhibits oxidative stress and inflammation in ischemic stroke through miR-155-5p/IRF2BP2 axis. Neurochem Res 48:1382–1394. 10.1007/s11064-022-03830-7 36460840

[B78] Zhang Y, Ning C, Zhou H, Yan Y, Liu F, Huang Y (2021a) Interleukin-1β, interleukin-6, and interleukin-17A as indicators reflecting clinical response to celecoxib in ankylosing spondylitis patients. Ir J Med Sci 190:631–638. 10.1007/s11845-020-02366-5 32955700

[B79] Zhang Y, Wang L, Li X, Geng J (2021b) Preliminary analysis of immunoregulatory mechanism of hyperhomocysteinemia-induced brain injury in Wistar-Kyoto rats. Exp Ther Med 21:483. 10.3892/etm.2021.9914 33790992 PMC8005698

[B80] Zhang Y, Wei W, Cheng N, Wang K, Li B, Jiang X, Sun S (2012) Hepatitis C virus-induced up-regulation of microRNA-155 promotes hepatocarcinogenesis by activating Wnt signaling. Hepatology 56:1631–1640. 10.1002/hep.25849 22610915

[B81] Zhang Z, Jia H, Gu T, Hu Q, Yu J, Zang D, Song N, Wang H (2018) RNA sequencing and bioinformatics analysis of the long noncoding RNA-mRNA network in colorectal cancer. J Cell Biochem 119:9957–9966. 10.1002/jcb.27319 30145796

[B82] Zhao J, Li L, Fang G (2020) Salvianolic acid A attenuates cerebral ischemia/reperfusion injury induced rat brain damage, inflammation and apoptosis by regulating miR-499a/DDK1. Am J Transl Res 12:3288–3301. 32774700 PMC7407710

[B83] Zhou KX, et al. (2021) Increased nuclear transporter KPNA2 contributes to tumor immune evasion by enhancing PD-L1 expression in PDAC. J Immunol Res 2021:6694392. 10.1155/2021/6694392 33728352 PMC7939744

[B84] Zhu F, et al. (2020) miR-155 antagomir protect against DSS-induced colitis in mice through regulating Th17/Treg cell balance by Jarid2/Wnt/β-catenin. Biomed Pharmacother 126:109909. 10.1016/j.biopha.2020.109909 32135463

[B85] Zingale VD, Gugliandolo A, Mazzon E (2021) MiR-155: an important regulator of neuroinflammation. Int J Mol Sci 23:90. 10.3390/ijms23010090 35008513 PMC8745074

