# Supplementary figures and images for: MicroRNA-155 Inhibition Activates Wnt/β-Catenin Signaling to Restore Th17/Treg Cell Balance and Protect against Acute Ischemic Stroke
Source: eNeuro. 2025 Feb 18;12(2):ENEURO.0347-24.2024. doi: 10.1523/ENEURO.0347-24.2024 (PMC11842039; doi:10.1523/ENEURO.0347-24.2024)

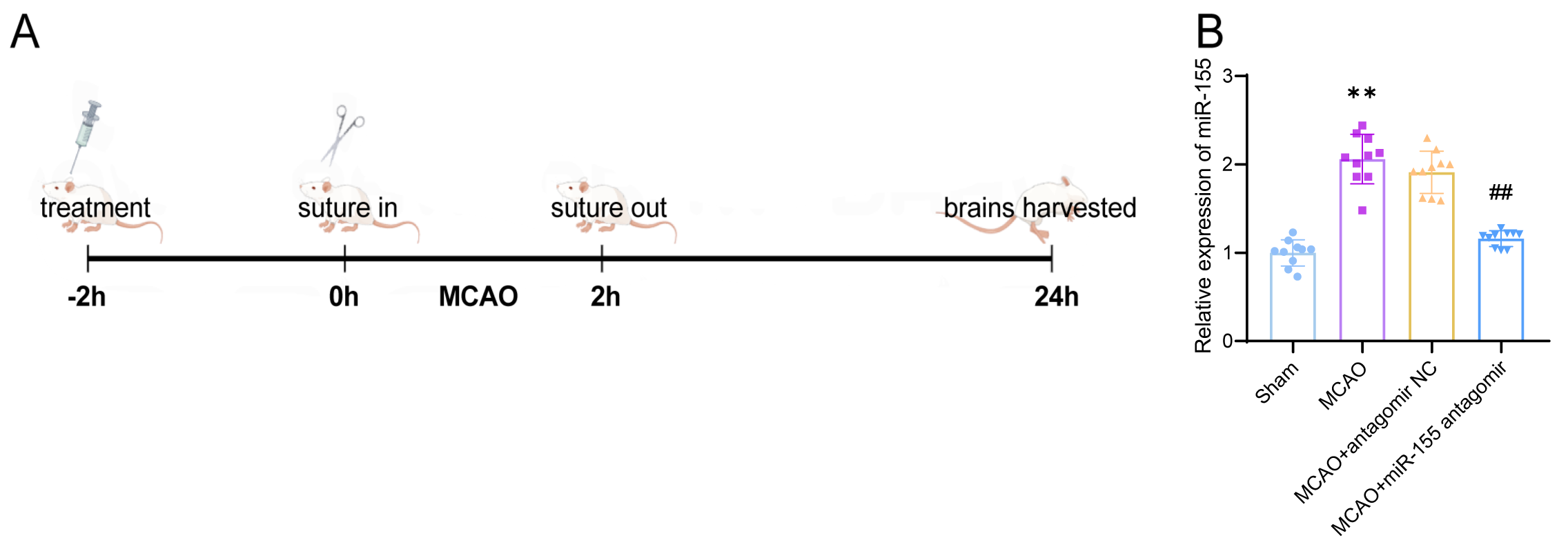

Supplement: Figure 3-1 — Construction of MCAO mouse model and detection of miR-155 expression levels. Note: (A) Pre-treatment of mice with miR-155 antagomir or antagomir NC via brain injection, followed by construction of MCAO model; (B) Detection of miR-155 expression levels in each group using qRT-PCR. ** indicates significant difference compared to the Sham group (P < 0.01); ## indicates significant difference compared to the MCAO + antagomir NC group (P < 0.01); n = 10. Download Figure 3-1, TIF file. [file eneuro-12-ENEURO.0347-24.2024-s002.tif]

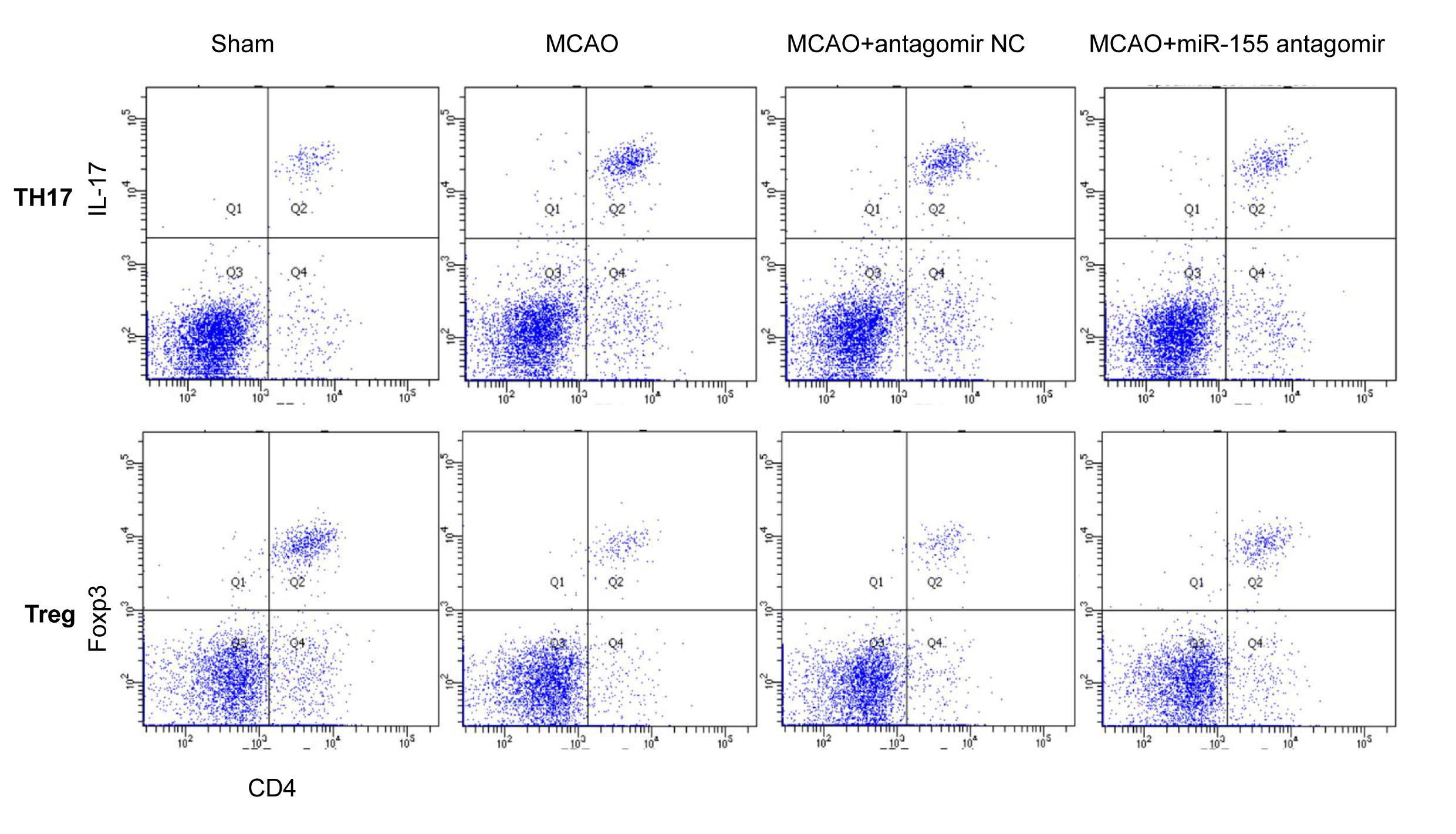

Supplement: Figure 4-1 — Analysis of changes in Th17 and Treg cells by flow cytometry. Note: Flow cytometry was used to detect the frequency of Th17 and Treg cells in peripheral blood. In the flow cytometry plot of Th17 cells, the x-axis represents FITC/CD4, and the y-axis represents PE/IL-17. In the flow cytometry plot of Treg cells, the x-axis represents FITC/CD4, and the y-axis represents PE/Foxp3. Download Figure 4-1, TIF file. [file eneuro-12-ENEURO.0347-24.2024-s003.tif]

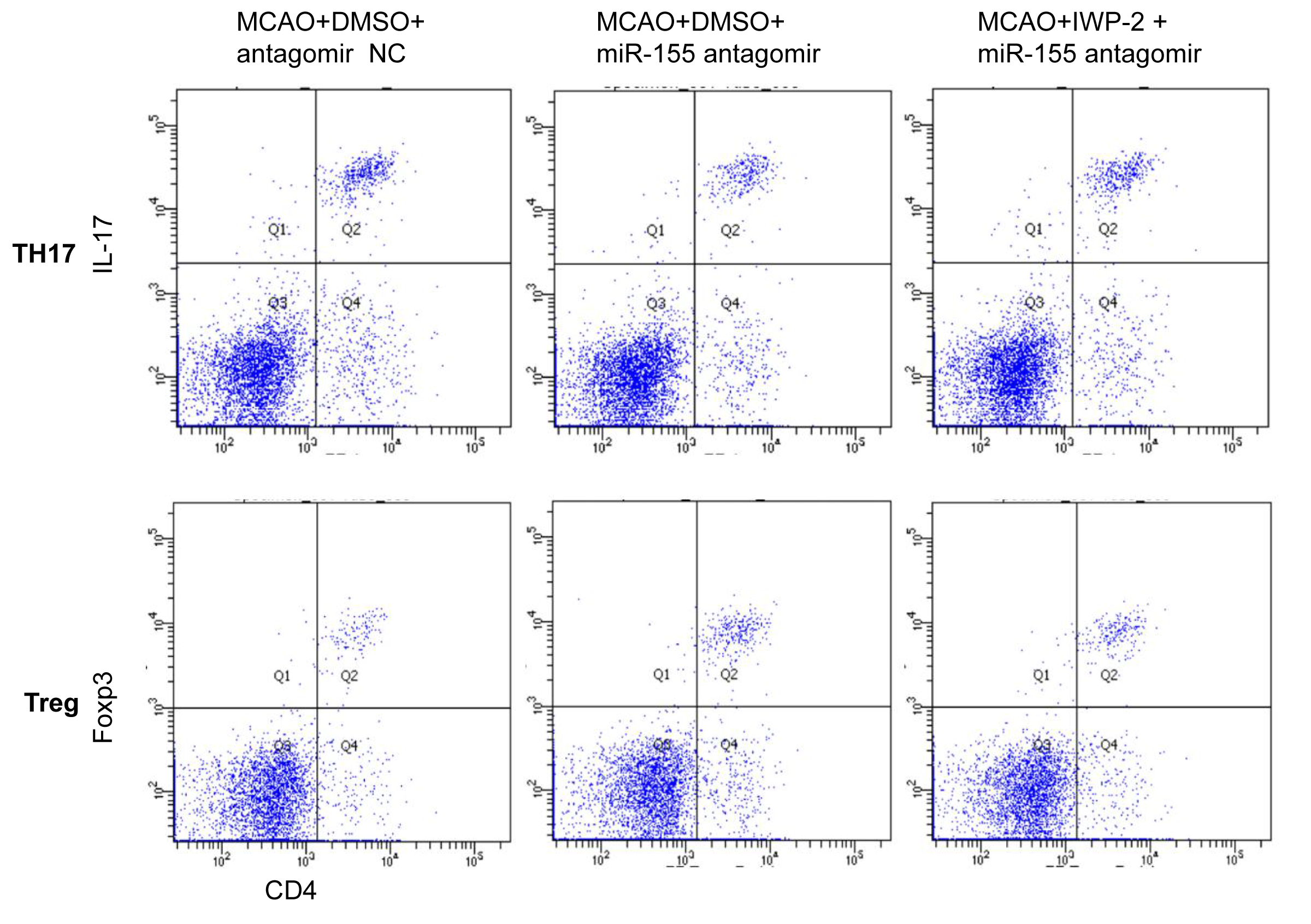

Supplement: Figure 5-1 — Analysis of changes in Th17 and Treg cells by flow cytometry. Note: Flow cytometry was used to detect the frequency of Th17 and Treg cells in peripheral blood. In the flow cytometry plot of Th17 cells, the x-axis represents FITC/CD4, and the y-axis represents PE/IL-17. In the flow cytometry plot of Treg cells, the x-axis represents FITC/CD4, and the y-axis represents PE/Foxp3. Download Figure, TIF file. [file eneuro-12-ENEURO.0347-24.2024-s004.tif]

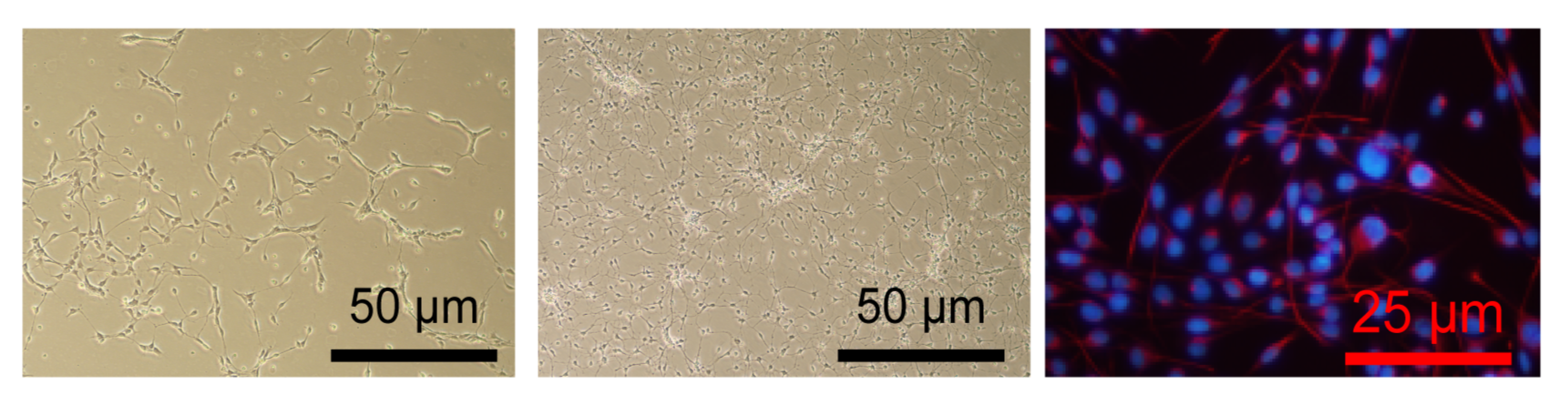

Supplement: Figure 6-1 — Morphological observation and identification of primary cortical neurons. Note: (A) Morphological image of neurons cultured for 2 days under an optical microscope; (B) Morphological image of neurons cultured for 7 days under an optical microscope; (C) Immunofluorescence staining for the expression of β-III Tubulin. β-III Tubulin is shown in red fluorescence, and the cell nucleus is marked with blue fluorescence. The scale bar is 50 μm. The cell experiment was repeated three times. Download Figure 6-1, TIF file. [file eneuro-12-ENEURO.0347-24.2024-s005.tif]
